# Programable Albumin-Hitchhiking Nanobodies Enhance the Delivery of STING Agonists to Potentiate Cancer Immunotherapy

**DOI:** 10.21203/rs.3.rs-3243545/v1

**Published:** 2024-05-08

**Authors:** John Wilson, Blaise Kimmel, Karan Arora, Neil Chada, Vijaya Bharti, Alexander Kwiatkowski, Jonah Finklestein, Ann Hanna, Emily Arner, Taylor Sheehy, Lucinda Pastora, Jinming Yang, Hayden Pagendarm, Payton Stone, Brandie Taylor, Lauren Hubert, Kathern Gibson-Corley, Jody May, John McLean, Jeffrey Rathmell, Ann Richmond, Wendy Rathmell, Justin Balko, Barbara Fingleton, Ebony Hargrove-Wiley

**Affiliations:** Vanderbilt University; Vanderbilt University; Vanderbilt University; Vanderbilt University; Vanderbilt University; Vanderbilt University; Vanderbilt University; Vanderbilt University Medical Center; Vanderbilt University Medical Center; Vanderbilt University; Vanderbilt University; Vanderbilt University Medical Center; Vanderbilt University; Vanderbilt University; Vanderbilt University Medical Center; Vanderbilt University; Vanderbilt University Medical Center; Vanderbilt University; Vanderbilt University Medical Center; Vanderbilt University Medical Center; Vanderbilt University Medical Center; Vanderbilt University Medical Center; Vanderbilt University Medical Center; Vanderbilt University

**Keywords:** STING, nanobody, albumin, cancer, immunotherapy, immune checkpoint blockade, adoptive T cell transfer

## Abstract

Stimulator of interferon genes (STING) is a promising target for potentiating antitumor immunity, but multiple pharmacological barriers limit the clinical utility, efficacy, and/or safety of STING agonists. Here we describe a modular platform for systemic administration of STING agonists based on nanobodies engineered for *in situ* hitchhiking of agonist cargo on serum albumin. Using site-selective bioconjugation chemistries to produce molecularly defined products, we found that covalent conjugation of a STING agonist to anti-albumin nanobodies improved pharmacokinetics and increased cargo accumulation in tumor tissue, stimulating innate immune programs that increased the infiltration of activated natural killer cells and T cells, which potently inhibited tumor growth in multiple mouse tumor models. We also demonstrated the programmability of the platform through the recombinant integration of a second nanobody domain that targeted programmed cell death ligand-1 (PD-L1), which further increased cargo delivery to tumor sites while also blocking immunosuppressive PD-1/PD-L1 interactions. This bivalent nanobody carrier for covalently conjugated STING agonists stimulated robust antigen-specific T cell responses and long-lasting immunological memory, conferred enhanced therapeutic efficacy, and was effective as a neoadjuvant treatment for improving responses to adoptive T cell transfer therapy. Albumin-hitchhiking nanobodies thus offer an enabling, multimodal, and programmable platform for systemic delivery of STING agonists with potential to augment responses to multiple immunotherapeutic modalities.

## Introduction

Immune checkpoint inhibitors (ICIs) targeting CTLA-4 and PD-1/PD-L1 have revolutionized the treatment of an increasing number of cancers but are still only effective for a relatively small fraction of patients (~ 15%).^1^ For many cancers, this can be attributed, in part, to poor tumor immunogenicity and an immunosuppressive (i.e., “cold”) tumor microenvironment (TME) that restricts the infiltration and/or function of antitumor T cells.^2, 3^ The innate immune system plays a critical role in cancer immune surveillance,^4^ with clinical evidence linking activation of certain pattern recognition receptor (PRR) signaling pathways to increased T cell infiltration and responses to ICIs in cancer patients.^5, 6^ Accordingly, the relationship between innate and adaptive antitumor immunity has motivated the clinical exploration and continued development of agonists targeting PRRs, including toll-like receptors (TLRs), RIG-I-like receptors (RLRs), and stimulator of interferon genes (STING). Activation of these pathways can induce a coordinated antitumor immune response by triggering the production of type-I interferons (IFN-Is), proinflammatory cytokines, chemokines, costimulatory molecules, and other mediators that potentiate T cell responses and enhance the efficacy of ICIs.^4, 7, 8^ PRR agonists have typically been administered intratumorally as an “*in situ* vaccine” with the intent to stimulate a systemic adaptive immune response that mediates distal tumor regression and/or immune memory to protect against disease recurrence.^2, 9^ While promising, intralesional therapy may not be feasible or practical for patients with metastatic, poorly accessible tumors, particularly for repeated dosing.^10^ Importantly, intratumoral administration has thus far yielded underwhelming outcomes in the clinic,^11^ motivating a need for systemically administered therapies targeting PRR agonists.

Amongst the PRRs, STING has emerged as one of the most promising targets for stimulating antitumor innate immunity,^12–14^ with remarkable efficacy in preclinical models leading to clinical trials of a growing arsenal of STING-activating therapeutics.^15, 16^ However, clinical exploration of STING agonists has been primarily restricted to intratumoral administration of cyclic dinucleotides (CDN) and, unfortunately, has yielded disappointing results.^17^ This can be partially attributed to both the aforementioned limitations of intratumoral administration as well as the poor drug-like properties of CDNs (i.e., anionic small molecules) that limit their activity and efficacy for systemic administration.^15^ This challenge has prompted the development of several promising nanoparticle-based drug carriers for CDNs^15, 18–22^ as well as small molecule STING agonists with improved chemical properties for systemic administration.^15, 23, 24^ However, therapeutic targeting of STING remains a considerable challenge owing to multiple intertwined pharmacological barriers, including suboptimal pharmacokinetics and poor tumor accumulation, that limit efficacy and increase the risk of inflammatory toxicities.^15, 25^ Hence, there is a need for drug delivery technologies that afford increased spatiotemporal control over the delivery of systemically administered STING agonists for the treatment of advanced and metastatic disease.

Here we present the development of a modular platform for safe and effective systemic administration of STING agonists based on the concept of “albumin-hitchhiking.”^26^ Albumin is a promising drug carrier based on its long circulation half-life and proclivity to accumulate at tumor sites via both passive and active transport mechanisms.^27–29^ Albumin and albumin-binding chaperones have been widely employed to improve the delivery of chemotherapeutics, exemplified by albumin-bound paclitaxel (Abraxane^®^)^30^, as well as protein^31^, peptide^32^, and nucleic acid therapeutics^30^. Inspired by this previous work that motivates the unexplored potential of albumin as a carrier for STING agonists, we engineered a high-affinity anti-albumin nanobody (i.e., single-domain antibody) for site-selective enzymatic bioconjugation of STING agonists via biorthogonal chemistry. Employing a conjugatable diamidobenzimidazole (diABZI) STING agonist as a clinically relevant example, we demonstrate that nanobody hitchhiking of STING agonists on serum albumin dramatically improves their pharmacological properties and increases tumor accumulation, leading to a reduction in tumor burden and improved therapeutic outcomes in multiple mouse tumor models. We further demonstrate the programmability of the platform for integrating tumor targeting and additional immunoregulatory functions through the development of a bispecific nanobody-diABZI conjugate that binds to both albumin and the immune checkpoint ligand PD-L1. We demonstrate that use of this bivalent nanobody carrier for STING agonist delivery further increases tumor accumulation while also inhibiting immunosuppressive PD-1/PD-L1 interactions, resulting in a reprograming of the tumor microenvironment (TME) to a more immunogenic “hot” milieu and a priming of antitumor T cells that further potentiate responses to multiple immunotherapeutic modalities. Collectively, our study positions albumin-hitchhiking, nanobody-STING agonist conjugates as an enabling, multimodal, and programmable platform for cancer immunotherapy with high translational potential.

## Results

### Synthesis of albumin-hitchhiking nanobody-STING agonist conjugates

**Figure 1:**
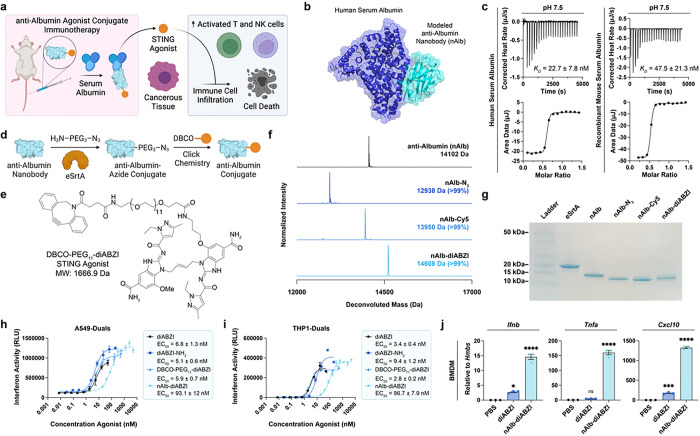
Design, synthesis, and *in vitro* characterization of an anti-albumin nanobody for site-selective conjugation of STING agonists. **(a)** Scheme depicting the concept of an albumin-hitchhiking nanobody-STING agonist conjugate for cancer immunotherapy. Anti-albumin nanobodies conjugated to STING agonists bind to circulating albumin *in situ*, resulting in improved pharmacokinetics and increased biodistribution to tumor sites that stimulates antitumor innate and adaptive immune responses. **(b)** Computational model of the anti-albumin nanobody (nAlb) binding at domain IIB of human serum albumin. **(c)** Isothermal calorimetry (ITC) traces (top) and binding isotherms (bottom) of nAlb binding to human and mouse serum albumin at pH 7.5. **(d)** Reaction scheme for generating molecularly homogeneous nAlb conjugates through site-selective enzymatic ligation of an amine-PEG_3_-azide followed by conjugation of agonist or dye cargo through copper-free click chemistry addition. **(e)** Structure of diABZI STING agonist conjugated to a DBCO-PEG_11_ handle for ligation to azide-functionalized nanobodies via click-chemistry. **(f)** Electrospray ionization mass spectrometry (ESI-MS) and **(g)** sodium dodecyl sulfate polyacrylamide electrophoresis (SDS-PAGE) demonstrating nanobody conjugate purity and molecular weight. **(h-i)** Dose-response curves in **(h)** A549-Dual (n=3) and **(i)** THP1-Dual IFN-I reporter cell lines (n=3) with estimated EC_50_ values indicated in the legends. **(j)** qPCR analysis of gene expression in murine bone marrow derived macrophages (BMDM) treated *in vitro* with 0.25 μM of free diABZI or nAlb-diABZI conjugate (n=3). *P* values determined by one-way ANOVA with post-hoc Tukey’s correction for multiple comparisons; *P≤0.05, **P≤0.01, ***P≤0.001, and ****P<0.0001 compared to PBS control. Replicates are noted as biological, and data shown as mean ± SEM.

We hypothesized that conjugation of a STING agonist to an albumin binding chaperone would extend blood circulation half-life and increase accumulation in cancerous tissue, enriching the production of cytokines and chemokines that facilitated the recruitment, proliferation, and activation of leukocytes to the TME, which promotes cancer cell death (Fig. 1a). While several promising albumin-binding molecules have been described, including small molecules, fatty acids, and peptides,^27, 29^ we elected to build our platform from a nanobody with high affinity for albumin because nanobodies are modular and programmable via genetic engineering, are molecularly well-defined, are amenable to scalable industrial manufacturing, and are components of approved and clinically-advanced therapeutics, including ozoralizumab, which contains an anti-albumin nanobody domain.^31^ Additionally, we sought to avoid the potential risk of accelerated albumin clearance that can occur due to direct covalent drug conjugation strategies^27, 29^ and to minimize the liver accumulation associated with the use of lipid-based albumin binders^30^, a challenge also faced by many promising nanoparticle-based STING agonists.^19, 21, 33, 34^ We therefore recombinantly expressed a previously described nanobody domain – termed nAlb – that binds with nanomolar affinity to serum albumin (Fig. 1b).^35^ We modeled the binding of the nanobody domain to human serum albumin (HSA) using RoseTTAFold to generate the nAlb nanobody and RosettaDock to predict the binding site of the nanobody to the serum protein albumin. We found that the nAlb nanobody reached an optimal energy conformation through binding at domain IIB of HSA, indicating that nAlb does not compete with albumin binding to FcRn, which facilities its long serum half-life (PDB: 4N0F). The binding affinity of nAlb was verified using isothermal calorimetry (ITC) both at physiological pH (7.5) and at endosomal pH (5.5), where nAlb maintained nanomolar affinity to both HSA and recombinant mouse serum albumin (rMSA) (Fig. 1c, **Fig. S1**).

To enable site-selective ligation of STING agonists, we cloned the C-terminal of the nAlb nanobody to present a selective ligation tag (LPETGGHHHHHHEPEA) that acts as a substrate for an engineered pentamutant of sortase A designed to selectively ligate any primary amine-containing small molecule to the C-terminal of a protein,^36^ offering high programmability in the design. Using this approach, we ligated an amino-PEG_3_-azide linker, which conferred a single azide functional handle on the nAlb nanobody and can be used to ligate cargo via strain-promoted azide-alkyne cycloaddition (Fig. 1d,f). While this strategy is amenable to ligation of diverse classes of STING agonists, we selected a diABZI compound since ongoing clinical trials are exploring similar agents as a systemically administered immunotherapy (e.g., NCT03843359). To enable covalent conjugation to the nanobody, we synthesized a diABZI variant that was functionalized with an azide-reactive DBCO group and a PEG_11_ spacer (DBCO-PEG_11_-diABZI) at the 7 position of one of the benzimidazole groups (Fig. 1e, **Fig. S2–4**), a modification that is not predicted to interfere with diABZI binding to STING. We then used copper-free click chemistry to install a single DBCO-PEG_11_-diABZI STING agonist or a DBCO-functionalized sulfo-Cy5 (referred to herein as Cy5) dye onto the nanobody and verified precise 1:1 conjugation by electrospray ionization mass spectrometry (ESI-MS) (Fig. 1f) and sodium dodecyl sulfate polyacrylamide electrophoresis (SDS-PAGE) (Fig. 1g).

We evaluated the activity of the nAlb conjugated STING agonist (nAlb-diABZI) as well as the parent DBCO-PEG_11_-diABZI compound and a previously optimized diABZI^23^ (compound 3; referred to henceforth as diABZI) in two human reporter cell lines for type-I interferon (IFN-I) production: monocytes (THP1-Dual) and lung carcinoma cells (A549-Dual) (Fig. 1h,i). We found that the DBCO-PEG_11_-diABZI variant retained a near identical EC_50_ value to the original diABZI agonist from literature, while, as expected, the *in vitro* activity of the nAlb-diABZI conjugate was reduced but nonetheless maintained high sub-100 nM activity for IFN-I production. Further, we tested the activity of the nAlb-diABZI conjugate in murine bone marrow derived macrophages (BMDM), demonstrating that nAlb-diABZI stimulated the expression of STING-driven cytokines *Ifnb1, Tnf*, and *CXCL10* after 4 hours (Fig. 1j).

### Albumin-hitchhiking nanobodies exhibit tumor tropism and enrich cargo delivery

**Figure 2:**
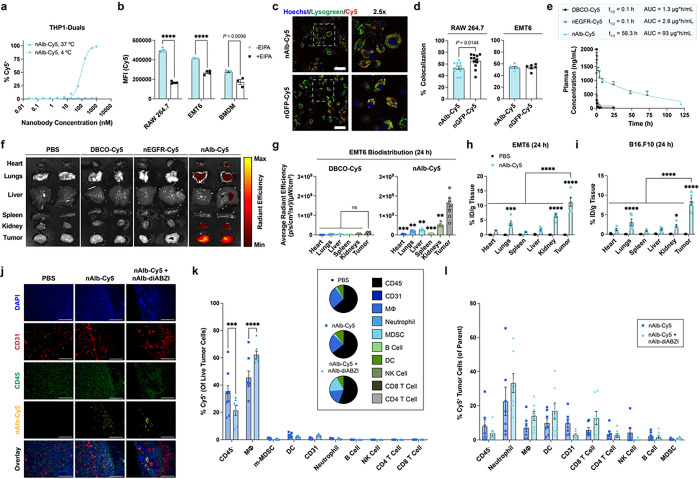
Anti-albumin nanobodies increase cargo delivery to tumor sites to promote uptake by cancer cells and tumor-associated myeloid cells. **(a)** Dose-response curve for nanobody-Cy5 conjugate surface binding and intracellular uptake at 37 °C and 4 °C measured by flow cytometry in EGFR^−^ (THP-1) *in vitro*. **(b)** Uptake of nAlb-Cy5 (2 μM) in RAW 264.7, EMT6, and BMDM cells with the addition of control PBS (−EIPA) or macropinocytosis inhibitor (+EIPA). **(c)** Colocalization of Cy5 (red) with lysotracker green (green) and Hoechst (blue) in RAW 264.7 cells with **(d)** percent colocalization determination for nAlb-Cy5 and nGFP-Cy5 in RAW 264.7 and EMT6 cells. (scale bar: 100 μm) **(e)** Pharmacokinetics of free DBCO-Cy5 dye and indicated nanobody-Cy5 conjugates injected intravenously at 2 mg/kg in healthy female C57BL/6 mice (n=5). Elimination phase half-life and area under the curve (AUC) are indicated in legend. **(f)** Representative IVIS fluorescent images of excised tumors and major organs and **(g)** quantification of average radiant efficiencies 24 h following intravenous administration of vehicle (PBS), DBCO-Cy5, nEGFR-Cy5, and nAlb-Cy5 at 2 mg/kg to female Balb/c mice with orthotopic EMT6 breast tumors (n=5–8). *P* values determined by one-way ANOVA with post-hoc Tukey’s correction for multiple comparisons; *P≤0.05, **P≤0.01, ***P≤0.001, and ****P<0.0001 compared to the tumor. **(h-i)** Quantification of percent injected dose per gram of tissue (% ID/g) 24 h following intravenous administration of vehicle (PBS) and nAlb-Cy5 at 2 mg/kg to **(h)** female Balb/c mice with orthotopic EMT6 breast tumors (n=5) and **(i)** female C57BL/6 mice with subcutaneous B16.F10 tumors (n=5). *P* values determined by two-way ANOVA with post-hoc Tukey’s correction for multiple comparisons; *P≤0.05, **P≤0.01, ***P≤0.001, and ****P<0.0001. **(j)** Representative fluorescent microscopy images of tumor sections stained for DAPI (blue), CD45 (green), and CD31 (red) 24 h following administration of nAlb-Cy5 (yellow) alone or in combination with nAlb-diABZI (scale bar: 200 μm). **(k-l)** Flow cytometric analysis of nAlb-Cy5 cellular uptake by in EMT6 tumors evaluated as **(k)** the percentage of indicated cell type comprising all Cy5^+^ live cells or **(l)** as the percentage of Cy5^+^ cells within an indicated live cell population 24 h following administration of vehicle (PBS), nAlb-Cy5 alone, or nAlb-Cy5 co-administered with nAlb-diABZI; median fluorescent intensities (MFI) for each cell population is shown in **Fig. S12** (n=7–8). Inset: percentage of indicated cell population in the tumor as measured by flow cytometry. DC: dendritic cell; Mj: macrophage; MDSC: myeloid derived suppressor cell; NK: natural killer cell. P values determined by one-way ANOVA with post-hoc Tukey’s correction for multiple comparisons; *P≤0.05, **P≤0.01, ***P≤0.001, and ****P<0.0001 compared to PBS control. Replicates are noted as biological, and data shown as mean ± SEM.

While still incompletely understood and variable across cancers and patients^29^, albumin can accumulate in tumor tissue through several interrelated mechanisms, including passive diffusion through leaky tumor vasculature, active transport via endothelial cell transcytosis, binding to SPARC (secreted protein acidic and rich in cysteine) produced by cancer cells, and cellular uptake and catabolism by cancer and tumor-associated immune cells such as macrophages.^27, 29^ Albumin has been reported to enter cancer cells and tumor-associated myeloid cells (e.g., macrophages) through both albumin-dependent, receptor-mediated pathways as well as by micropinocytosis.^27, 29^ Though mechanisms of cellular albumin internalization may vary between tumor and cell types, we sought to gain insight into how nAlb-diABZI enters cells and activates STING. First, we first validated that intracellular uptake of nAlb-Cy5 was abrogated at 4°C indicating an active endocytotic mechanism (Fig. 2a, **Extended Data** Fig. 1); by contrast diABZI can enter cells by passive transport across the plasma membrane.^23^ We next assessed if albumin binding enhanced nAlb internalization by EMT6 and myeloid cells. To test this, we first used flow cytometry compare the cellular uptake of nAlb-Cy5 to a negative control nanobody targeting GFP, nGFP-Cy5 (**Fig. S5**), in serum containing media, finding insignificant or minor differences in cellular uptake between nAlb-Cy5 and nGFP-Cy5 (**Extended Data** Fig. 1). While eliminating serum from culture media decreased nAlb-Cy5 uptake, this occurred to the same extent for nGFP-Cy5, again indicating that cellular uptake occurs predominantly in an albumin receptor-independent manner in these cell types. Albumin can also be internalized by cancer and tumor-associated immune cell populations through non-receptor-mediated micropinocytosis. To evaluate this, we inhibited micropinocytosis in EMT6 cells, RAW264.7 macrophages, and BMDMs using 5-[N-ethyl-N-isopropropyl] amiloride (EIPA), which significantly reduced nAlb-Cy5 uptake (Fig. 2b). Given that macropinosomes often traffic to lysosomes, we next assessed colocalization of nAlb-Cy5 with lysotracker and found that a substantial and similar fraction (> 50%) of nAlb-Cy5 and nGFP-Cy5 was colocalized with lysosomes or late endosomes in EMT6 and RAW264.7 cells (Fig. 2c,d). As expected, nAlb-diABZI did not mediate endosomal disruption as assessed using a previously described galectin 8 (Gal8) endosomal recruitment assay (**Extended Data** Fig. 1)^41^.

To gain insight into how amide-linked diABZI is released from the nanobody upon cellular internalization, we incubated nAlb-diABZI with lysosomes isolated from rat liver (Tritosomes), which are used to investigate stability and catabolism of molecules trafficked to an endosome-lysosome pathway, and used MALDI mass spectroscopy to assess the emergence of a PEGylated diABZI adduct that would be predicted due to amide bond cleavage by lysosomal proteases (**Fig. S6**). We observed the presence of this peak as early as 1 h following incubation with Tritosomes, suggesting that a fraction of nAlb-diABZI is lysosomally degraded to release a PEGylated diABZI variant. We synthesized this compound (**Fig. S7–9**) and evaluated *in vitro* activity in THP1 IFN-I reporter cells, finding that it had a similar EC_50_ value to the previously described diABZI molecule, which can enter cells through passive transport^23^ (**Fig. S10**). While *in vivo* mechanisms of albumin transport and cellular uptake are complex and still not fully understood^29^, taken together our data suggests that nAlb that accumulates at tumor sites is macropinocytosed, primarily by tumor-associated myeloid cells, resulting in lysosomal degradation and release of a diABZI variant that activates STING.

We next evaluated the pharmacokinetics and biodistribution of nAlb site-selectively conjugated to Cy5 as described for diABZI above (nAlb-Cy5) compared to an analogous control anti-EGFR nanobody (nEGFR) that we cloned and Cy5-labeled using the same strategy (**Fig. S5**). To assess the pharmacokinetic profile achieved by using anti-albumin nanobody hitchhiking, we intravenously (I.V.) administered free DBCO-Cy5, nEGFR-Cy5, and nAlb-Cy5 in healthy female C57BL/6 mice and collected blood at discrete time points over several days (Fig. 2e). By measuring the concentration of Cy5 in the serum using fluorescence spectroscopy, we determined the elimination half-life of both the free dye and the nEGFR-Cy5 conjugate to be approximately 5 minutes, matching the expected half-life of a typical nanobody that is rapidly cleared via renal excretion due to its small size (~ 15kDa).^37^ However, the nAlb-Cy5 conjugate had an elimination half-life of approximately 55 hours, consistent with *in situ* binding to and hitchhiking on serum albumin, which has a half-life of ~ 35–40 h in mice.^38^ By comparison the reported half-life of diABZI is ~ 90 minutes,^23^ while that of CDNs is typically < 5 min.^33^ We next tracked the biodistribution of DBCO-Cy5, nEGFR-Cy5, and nAlb-Cy5 in female Balb/c mice with orthotopic EMT6 (EGFR^+^) breast tumors inoculated in the mammary fat pad. At 24 hours post-administration, mice were euthanized, and major organs and tumors were imaged with an *in vivo* imaging system (IVIS) instrument to evaluate Cy5 biodistribution (Fig. 2f,g) and tissue was homogenized for quantification of Cy5 using fluorescent spectroscopy (Fig. 2h). We observed minimal Cy5 fluorescence in major organs for both nEGFR-Cy5 and nAlb-Cy5 conjugates, but substantial tumor accumulation of only the nAlb-Cy5 conjugate, corresponding to ~ 11% injected dose/gram tissue, significantly higher than other organs; similar findings were observed in a B16.F10 tumor model (Fig. 2i). Immunofluorescent staining of excised and cryosectioned tumors (Fig. 2j) further confirmed nAlb-Cy5 accumulation at the tumor site, with the highest Cy5 fluorescence observed proximal to CD31^+^ tumor vasculature and with Cy5 signal also observed within the tumor stroma (e.g., colocalizing with CD45^+^ immune cells). Albumin binding to SPARC expressed in tumor tissue has also been implicated in increased accumulation of albumin-binding therapeutics,^27^ and we found that SPARC is expressed in both EMT6 and B16.F10 tumors (**Fig. S11**) and may therefore contribute to nAlb accumulation.

Based on the significant tumor accumulation of nAlb-Cy5, we next used flow cytometry to determine which tumor-associated cell populations internalized the conjugate (Fig. 2k,l, **Fig. S12**). At 24 h after I.V. injection of nAlb-Cy5, we found that ~ 8% of all live cells in the tumor were Cy5^+^ (**Fig. S13**) and, amongst Cy5^+^ cells, the majority were CD45^−^CD31^−^ cells, which are primarily cancer cells, and tumor-associated CD11b^+^F4/80^+^ macrophages (Fig. 2k). Cancer cells (CD45^−^CD31^−^) and macrophages are the most prevalent cell populations in the EMT6 tumor model and have been reported to endocytose albumin in tumors.^39, 40^ Evaluating nAlb-Cy5 uptake within specific cell populations, we found that ~ 5–10% of cancer cells (CD31^−^CD45^−^), macrophages (CD11b^+^F4/80^+^), and dendritic cells (CD11c^+^) were Cy5^+^ with a higher (~ 15–20%) frequency of Cy5^+^ CD45^−^CD31^+^ endothelial cells and neutrophils (Fig. 2l). As assessed by Cy5 median fluorescent intensity (MFI), the cell populations with the highest degree of nAlb-Cy5 uptake were CD45^−^CD31^+^ endothelial cells, neutrophils, dendritic cells, macrophages, and cancer (CD45^−^CD31^−^) cells (**Fig. S12**). To determine if this cellular uptake profile was influenced by STING activation, we concurrently administered nAlb-Cy5 with nAlb-diABZI and found that the addition of nAlb-diABZI primarily impacted the myeloid cell composition of the tumor at 24h, resulting in an increased frequency of neutrophils and MDSCs and a reduction in macrophages (Fig. 2k, **inset**) while slightly biasing nAlb-Cy5 uptake towards macrophages, dendritic cells, and neutrophils. We also evaluated cellular uptake of nAlb-Cy5 in the spleen (**Fig. S12**), which, while not a major organ of distribution for nAlb-Cy5, is a potentially important secondary lymphoid organ for generating systemic antitumor immunity, finding that ~ 5–10% of macrophages and dendritic cells were Cy5^+^. Taken together, these data demonstrate that nanobody albumin hitchhiking can increase tumor accumulation to allow for endocytosis of cargo by multiple tumor-associated cell types.

### nAlb-diABZI potently stimulates STING activation in the TME to inhibit tumor growth

Based on the ability of nAlb to enhance cargo distribution to tumor sites, we next performed a dose-response response study to evaluate the therapeutic efficacy of nAlb-diABZI conjugates in an established non- or low-immunogenic (immunologically “cold”) B16.F10 tumor model that is resistant to ICB (**Fig. S14**).^42^ Using a treatment regimen that we and others have employed for evaluation of STING agonists,^33, 43^ we intravenously administered nAlb-diABZI to mice bearing ~ 75 mm^3^ B16.F10 tumors at doses ranging from 5–0.05 μg diABZI content, finding that all doses significantly (*P* < 0.0001) inhibited tumor growth and extended survival time. Notably, the 5 μg dose significantly (*P* < 0.0001) enhanced efficacy relative to a 3x higher dose of diABZI, demonstrating the enhancement in potency enabled through albumin-hitchhiking. While the 5 μg dose resulted in ~ 10–12% weight loss, this was transient and occurred only after the first injection (**Fig. S14a**). Nonetheless, towards maximizing the safety profile of the treatment, we selected a dose of 1.25 μg, confirmed antitumor efficacy of both a single and three-dose regimen in the B16.F10 model (**Fig. S14d-g; Fig. S15**), and performed a preclinical analysis of nAlb-diABZI toxicity (**Extended Data** Fig. 2). Healthy mice were administered vehicle (PBS) or nAlb-diABZI (1.25 μg diABZI) intravenously three times spaced three days apart, weight loss was monitored daily, and blood was collected 4 and 24 hr after the first injection for analysis of serum cytokines (**Extended Data** Fig. 2). In response to nAlb-diABZI, mice experienced only a mild (~ 5%) and transient weight loss similar to that described for nanoparticle-based delivery of STING agonists^18, 19, 21^ with elevated plasma levels of STING-driven cytokines with antitumor functions (e.g., type I IFN, IL-12 4 h following injection, which returned to near baseline by 24 h. Mice were euthanized a week following the last injection, blood was collected for biochemistry analysis (**Extended** Fig. 2d), and major organs were isolated for histological evaluation (**Extended** Fig. 2e) by a board certified veterinary pathologist, who observed no clinically significant changes between the untreated control mice and nAlb-diABZI treated mice, consistent with insignificant or minor changes in blood biochemistry and cellular composition. Based on this favorable safety profile at a therapeutically effective dose in a challenging B16.F10 tumor model, we selected a dose of 1.25 μg for all subsequent studies.

**Figure 3:**
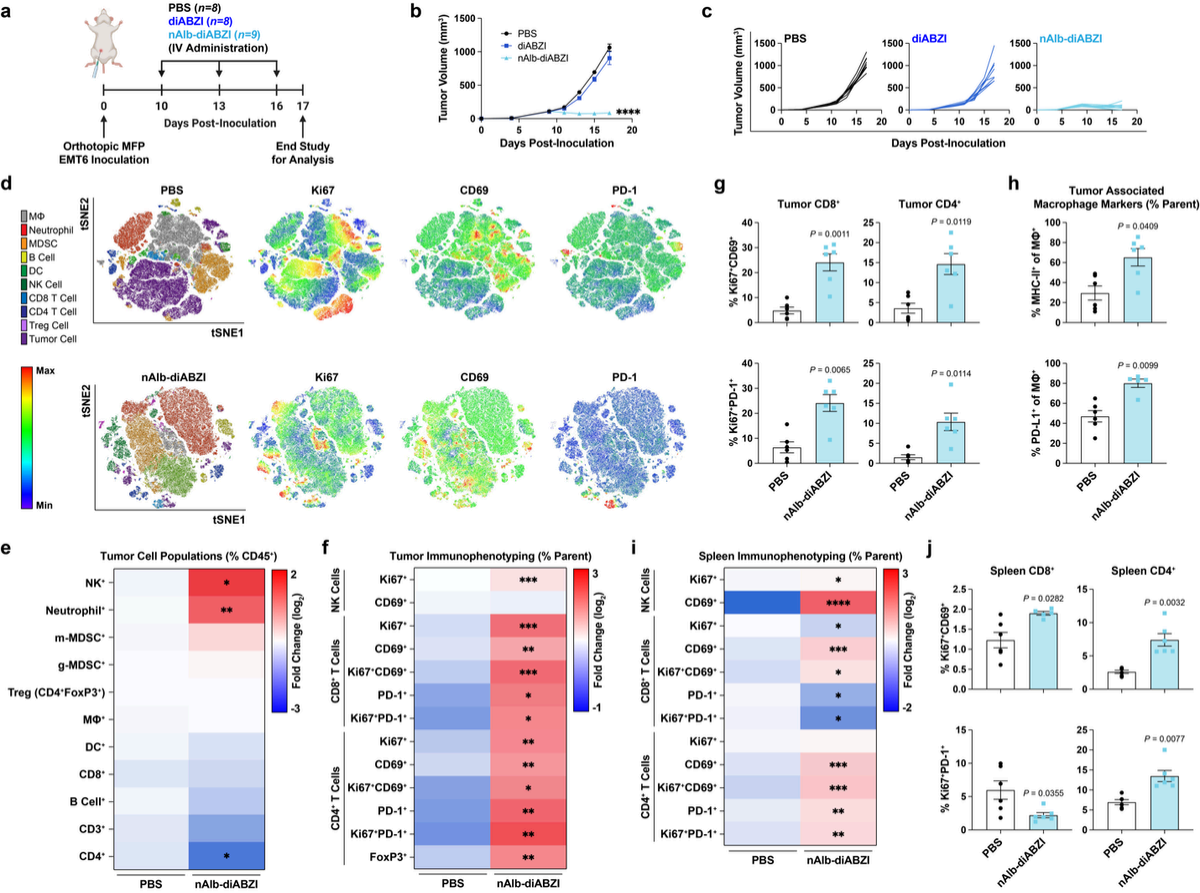
Albumin-hitchhiking STING agonist inhibits breast tumor growth by shifting the immunocellular profile of the TME. **(a)** Schematic of EMT6 tumor inoculations, treatment schedule, and study end point for gene expression and flow cytometry analysis. **(b)** Tumor growth curves, and **(c)** spider plots of individual tumor growth curves for each mice with EMT6 tumors treated as indicated (n=8–9). SEM with *P* value determined by two-way ANOVA with post-hoc Tukey’s correction for multiple comparisons; ****P<0.0001 on day 17 for all groups compared to PBS. **(d-j)** Flow cytometric analysis of breast tumors and spleen 24 h following final dose of nAlb-diABZI. (d) tSNE plots of live cells in EMT6 tumors colored by cell population with relative expression level of Ki67, CD69, and PD-1 as indicated on heat map. DC: dendritic cell; Mj: macrophage; NK: natural killer cell; MDSC: myeloid-derived suppressor cell. **(e-f)** Heat maps summarizing **(e)** the fold change in the percentage of indicated cell population and **(f)** fold change in the frequency of NK cells, CD8^+^ T cells, and CD4^+^ T cells expressing the indicated marker or marker combination in EMT6 breast tumors. **(g)** Quantification of Ki67^+^CD69^+^ and Ki67^+^PD1^+^ CD8^+^ and CD4^+^ T cells in EMT6 tumors following treatment with vehicle (PBS) or nAlb-diABZI. **(h)** Quantification of frequency of MHC-II^+^ and PD-L1^+^ macrophages in EMT-6 tumors following treatment with vehicle (PBS) or nAlb-diABZI. **(i)** Heat map summarizing fold change in the frequency of NK cells, CD8^+^ T cells, and CD4^+^ T cells expressing activation markers within splenic populations. *P≤0.05, **P≤0.01, ***P≤0.001, and ****P<0.0001 indicate a statistically significant difference for heat-maps between PBS and AP-diABZI treated groups as determined by two-way ANOVA. **(j)** Quantification of Ki67^+^CD69^+^ and Ki67^+^PD1^+^ CD8^+^ and CD4^+^ T cells in spleens. *P≤0.05, **P≤0.01, ***P≤0.001, and ****P<0.0001 indicate a statistically significant difference between PBS and nAlb-diABZI treated groups as determined by Student’s t-test, n = 6 per group. Replicates are noted as biological, and data shown as mean ± SEM.

Given the significant tumor accumulation of nAlb observed in orthotopic EMT6 breast tumors – and considering that only approximately 20% of breast cancer patients benefit from PD-1/PD-L1 ICB^44^ – we next evaluated the capacity of nAlb-diABZI to create a TME that inhibited tumor growth. Female Balb/c mice were inoculated with EMT6 cells in a mammary fat pad (MFP) and treated with nAlb-diABZI, free diABZI, or vehicle (PBS) at a tumor volume of ~ 75 mm^3^ (Fig. 3a). Interestingly, treatment with nAlb-diABZI strongly suppressed tumor growth whereas the free diABZI STING agonist did not confer a therapeutic benefit (Fig. 3b,c). Consistent with accumulation of nAlb at tumor sites, we found a notable increase in the expression of genes associated with STING pathway activation, including *Ifnb1, Cxcl10*, *Cxcl9*, and *Tnfa* (**Fig. S16**).

To gain insight into the immunological mechanisms by which nAlb-diABZI inhibited tumor growth, we used multispectral flow cytometric immunophenotyping to quantify changes in key myeloid and lymphocyte populations and their phenotypes (Fig. 3d–j, **Extended Data** Fig. 3) in EMT6 tumors and in the spleen 24 h following the third nAlb-diABZI administration. We found that administration of nAlb-diABZI increased the infiltration of CD8^+^ T cells with considerably elevated markers of activation (CD69) and proliferation (Ki67) – as well as the frequency of Ki67^+^PD-1^+^ CD8^+^ T cells – which have been correlated with favorable responses to immunotherapy in patients.^45^ While there was a reduction in the overall frequency of CD4^+^ T cells this was associated with an increased frequency of CD69^+^Ki67^+^ and Ki67^+^PD-1^+^ CD4^+^ T cells. There was also a significant increase in the frequency of NK cells and Ki67^+^ NK cells in the TME; interestingly, the levels of splenic CD69^+^ and Ki67^+^ NK cells were also elevated, potentially suggesting mobilization of NKs from the spleen to the tumor (**Fig. S17**).^46^ Trends towards increased frequency of MDSCs (Fig. 3d,e), a significant increase in the frequency of FoxP3^+^CD4^+^ regulatory T cells (Fig. 3e,f), and elevated MHC-II and PD-L1 on macrophages (Fig. 3g,h) was also observed. Similar findings have been described for other STING agonists, which may act as counter regulatory mechanisms that may contribute to resistance to nAlb-diABZI as a monotherapy. In particular, MDSCs have been reported to reduce the efficacy of some STING-activating therapies^47–49^ and we therefore evaluated nAlb-diABZI in combination with orally administered SX-682, which inhibits CXCR1/2 chemokine receptors involved in MDSC recruitment^50^, but, surprisingly, found that SX-682 tended to reduce nAlb-diABZI efficacy (**Fig. S18**). We also used anti-Gr1 antibodies to deplete MDSCs (primarily gMDSCs)^51^ and again found a modest reduction in nAlb-diABZI efficacy (**Fig. S19**). Similar findings have been reported by others,^52^ reflecting the potentially complex roles of MDSCs in response to immunotherapy given their capacity to differentiate into mature antitumor effectors. Nonetheless, our data suggests that MDSCs do not strongly restrict the efficacy of nAlb-diABZI, at least in the EMT6 breast tumor model.

In addition to immunological resistance mechanisms, the efficacy of nAlb-diABZI may also be inhibited through generation of anti-nAlb or anti-diABZI antibodies that may lead to accelerated blood clearance.^53^ While albumin has been described to generate immune tolerance to antigenic cargo^54^ and nanobodies typically have low immunogenicity^55^, we addressed this possibility by intravenously administering healthy wild-type C57BL/6 mice with nAlb-diABZI on day 0, 3, and 6 and evaluated anti-VHH antibody titer in serum on day 14 and also compared the plasma half-life of nAlb-Cy5 to untreated mice. We did not detect an anti-VHH antibody response in serum (**Fig. S20**) and observed a similar nAlb-Cy5 half-life between untreated mice (~ 59 h) and treated mice (~ 64 h) (**Fig. S21**), suggesting that antibody-mediated nanobody clearance was unlikely to reduce nAlb-diABZI efficacy in our studies; however, this possibility cannot be discounted in humans where dose and treatment regimen, amongst other variables, will be different and therefore will need to be further investigated.

### Engineering an albumin-binding, bivalent nanobody fusion for combined STING agonist delivery and immune checkpoint inhibition

**Figure 4:**
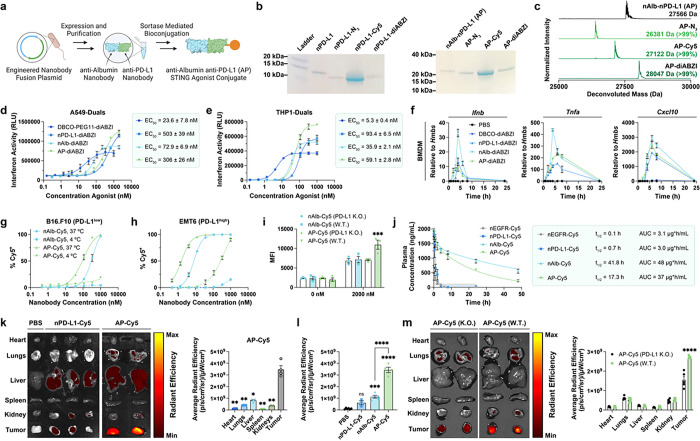
Design, synthesis, and testing of bivalent nanobody-STING agonist conjugate for albumin-hitchhiking and targeting of PD-L1. **(a)** Scheme for the cloning, expression, and bioconjugation of small molecule cargo to generate the AP-diABZI conjugate. **(b)** SDS-PAGE and **(c)** ESI-MS confirming the purity and molecular weight of AP conjugates. (d-e) Dose-response curves for indicated nanobody-diABZI conjugate in **(d)** A549-Dual (n=3) and **(e)** THP1-Dual IFN-I reporter cell lines (n=3) with estimated EC_50_ values indicated in the legends. **(f)** qPCR analysis of genes associated with STING activation in bone marrow derived macrophages (BMDMs) in response to treatment at discrete time points (0, 0.5, 1, 2, 3, 4, 6, 8, 24 h) with indicated agonist at 0.25 μM (n=3). *P* values determined by one-way ANOVA with post-hoc Tukey’s correction for multiple comparisons; *P≤0.05, **P≤0.01, ***P≤0.001, and ****P<0.0001 compared to PBS control. **(g-h)** Dose response curve for nAlb-Cy5 and AP-Cy5 conjugate surface binding and intracellular uptake at 37 °C and 4 °C measured by flow cytometry in **(g)** B16.F10 cells (n=2–3) and **(h)** EMT6 cells (n=3). **(i)** Mean fluorescent intensity (MFI) for nAlb-Cy5 and AP-Cy5 conjugate surface binding at 2 μM compared to PBS (0 μM) for EMT6 W.T. and EMT6 PD-L1 K.O. cell lines at 37 °C. **(j)** Pharmacokinetics of indicated nanobody-Cy5 conjugate in healthy Balb/c female mice (n=5). Elimination phase half-life and area under the curve (AUC) are indicated in legend. **(k)** Representative IVIS fluorescent images of excised tumors and major organs (left) and quantification of average radiant efficiencies (right) of tumors and major organs 48 h after administration of nPD-L1-Cy5 and AP-Cy5 in mice with EMT6 breast tumors (n=3–4). *P* values determined by one-way ANOVA with post-hoc Tukey’s correction for multiple comparisons; *P≤0.05, **P≤0.01, ***P≤0.001, and ****P<0.0001 compared to the tumor group. **(l)** Comparison of Cy5 radiant efficiencies in tumor tissue 48 h following administration of indicated nanobody-Cy5 conjugate. P values determined by one-way ANOVA with post-hoc Tukey’s correction for multiple comparisons; *P≤0.05, **P≤0.01, ***P≤0.001, and ****P<0.0001 compared to the PBS control, as well as between nAlb-Cy5 and AP-Cy5. **(m)** Representative IVIS fluorescent images of excised tumors and major organs (left) and quantification of average radiant efficiencies (right) of tumors and major organs 48 h after administration of AP-Cy5 in mice with EMT6 W.T. and EMT6 PD-L1 K.O. breast tumors (n=5). *P* values determined by one-way ANOVA with post-hoc Tukey’s correction for multiple comparisons; *P≤0.05, **P≤0.01, ***P≤0.001, and ****P<0.0001 compared to the tumor group. Replicates are noted as biological, and data shown as mean ± SEM.

Having demonstrated the potent antitumor effects of our albumin hitchhiking STING agonist, we next sought to leverage the modularity of nanobody engineering to confer additional immunotherapeutic functionality and demonstrate the programmability of the platform. As a translationally-relevant example, we introduced a second previously described nanobody domain that binds to PD-L1 (anti–programmed cell death ligand 1).^56^ Our rationale for selecting PD-L1 was two-fold. First, we, and others, have demonstrated synergy between STING agonists and PD1/PD-L1 ICB in suppressing tumor growth, including evidence that STING activation can directly upregulate PD-L1 expression.^43, 57^ Second, PD-L1 can be expressed by both cancer cells and immunosuppressive myeloid cells in solid tumors,^58^ providing a molecular target for increasing tumor accumulation; indeed, anti-PD-L1 nanobodies have been employed previously in imaging applications with high selectivity for tumor tissue.^56^ We therefore hypothesized that an anti-albumin/anti-PD-L1 nanobody fusion would increase tumor targeting, while inhibiting immunoregulatory PD1/PD-L1 interactions that restrain responses to STING agonists. Thus, we generated a fusion protein that uses a genetic linker to connect both nanobody domains and maintained the C-terminal sortase ligation tag to generate an anti-albumin/anti-PD-L1 (AP)-STING agonist conjugate, termed AP-diABZI (Fig. 4a). We characterized the synthesis and generation of both anti-PD-L1 nanobody (nPD-L1) and AP conjugates to Cy5 and diABZI, showing that a single, homogeneous product that contained all three functional elements was formed (Fig. 4b–c, **Fig. S1, Fig. S10**). The *in vitro* activity of nPD-L1-diABZI and AP-diABZI was tested in A549-Dual and THP1-Dual IFN-I reporter cells (Fig. 4d,e) and by qPCR for analysis of STING-associated cytokines/chemokine gene expression in primary BMDMs and BMDCs (Fig. 4f, **Fig. S22**). We found that all nanobody-diABZI conjugates were potently active in both reporter cell lines without evidence of cytotoxicity (**Fig. S23**) and that nanobody-diABZI conjugates were more active than the parent DBCO-diABZI in BMDMs and triggered STING-associated gene expression with similar kinetics (Fig. 4f); both nAlb-diABZI and AP-diABZI were also active in murine bone marrow-derived dendritic cells (BMDC; **Fig. S22**). Additionally, we showed using flow cytometry that the incorporation of the PD-L1 targeting domain enhanced binding and internalization in B16.F10 (PD-L1^low^) and EMT6 (PD-L1^high^) cells (Fig. 4g–h) relative to the albumin binding nanobody domain alone, which we further confirmed by comparing internalization by wild-type and PD-L1 KO EMT-6 cells (Fig. 4i).

We next tested the hypothesis that integrating a PD-L1 binding domain would increase tumor accumulation. We administered 2 mg/kg of Cy5-conjugated nEGFR, nPD-L1, nAlb, and AP nanobodies to healthy Balb/c mice I.V. and collected blood at discrete time points to evaluate pharmacokinetics (Fig. 4j). We also administered Cy5-conjugated nanobodies to mice with orthotopic EMT6 breast tumors and euthanized mice at 48 h to quantify nanobody-Cy5 conjugate biodistribution to major organs and tumors using IVIS (Fig. 4k–l). While the AP-Cy5 conjugate had a shorter elimination half-life than nAlb-Cy5 (17 h to 55 hours, respectively), likely due to binding of target PD-L1 in tissue and removal from circulation, both carriers maintained an increased elimination half-life and AUC relative to either targeted nanobody (nEGFR and nPD-L1) alone, which were cleared rapidly from circulation (Fig. 4i). While AP is approximately twice the size (~ 28 kDa) of the anti-PD-L1 nanobody, both are below the threshold for renal clearance^37^ and, therefore, the increased circulation time of AP can be primarily attributed to the albumin hitchhiking functionality. Further, while the nPD-L1-Cy5 conjugate was observed at similarly low levels in major organs (liver and kidneys) and the tumor at 48 h (Fig. 4j), the AP-Cy5 conjugate demonstrated significant tumor accumulation (corresponding to 2.19 ± 0.43%ID/gram tumor) relative to major organs (Fig. 4k) and significant increase over nAlb alone (Fig. 4l–m). To further demonstrate increased tumor targeting, we compared the relative tumor accumulation of AP-Cy5 in breast tumors established using parental or PD-L1 knockout EMT-6 cells and found a significant decrease in tumor accumulation in the PD-L1 knockout model (Fig. 4m). It should be noted that PD-L1 was only knocked out of cancer cells in this model and that infiltrating myeloid cells can also express PD-L1 which may explain the modest < 2-fold decrease in AP-Cy5 accumulation. Nonetheless, these studies support our hypothesis that integrating a PD-L1 binding domain further improves delivery to tumor tissue.

### AP-diABZI reprograms the TME to eliminate breast tumors and generate immunological memory that prevents recurrent disease

**Figure 5:**
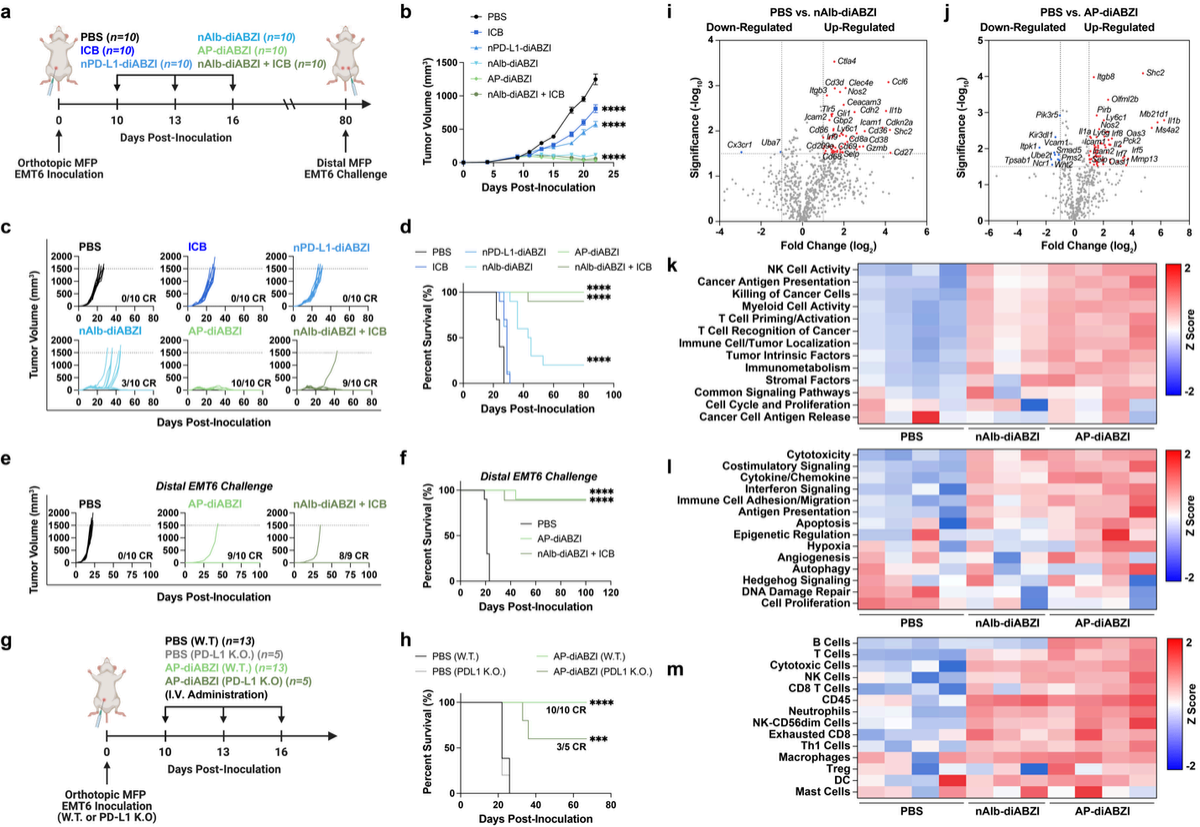
Systemic administration of AP-diABZI conjugates enhance antitumor immune and therapeutic responses in EMT6 breast cancer model. **(a)** Schematic of EMT6 tumor inoculation and treatment schedule (n=10). Anti-PD-L1 IgG (ICB) was injected I.P. at 100 μg and all nanobodies were injected I.V. at 1.25 μg of diABZI per injection. **(b)** Tumor growth curves, **(c)** spider plots of individual tumor growth curves, and **(d)** Kaplan-Meier survival plots for mice with EMT6 tumors treated as indicated. CR = complete responder; SEM with *P* value determined by two-way ANOVA with post-hoc Tukey’s correction for multiple comparisons; ****P<0.0001 on day 22 for all groups compared to PBS. Endpoint criteria of 1500 mm^3^ tumor volume with *P* value was determined by log-rank test; ****P<0.0001 compared to PBS control. **(e)** Spider plots of individual tumor growth curves and **(f)** Kaplan-Meier survival curves of mice challenged or re-challenged (complete responders after first treatment regimen) with EMT6 cells (n=9–10). **(g)** Scheme of EMT6 W.T. and EMT6 PD-L1 K.O. tumor inoculation and treatment schedule (n=5–13). AP-diABZI was injected I.V. at 1.25 μg of diABZI. **(h)** Kaplan-Meier survival plots for mice with EMT6 W.T. or PD-L1 K.O. tumors treated as indicated. **(i-j)** Volcano plots representing significance (−log_10_) and fold change (log_2_) for gene expression analysis in **(i)** nAlb-diABZI vs. PBS (n=4) and **(j)** AP-diABZI vs. PBS (n=4). **(k-m)** Heat maps of NanoString gene cluster matrices showing Z score fold changes for **(k)** functional gene annotations, **(l)** biological signatures, and **(m)** cell types. Replicates are noted as biological, and data shown as mean ± SEM.

We next investigated the anti-tumor effects of systemically administered AP-diABZI fusion in the orthotopic EMT6 tumor model, comparing effects to those elicited by the constitutive components nAlb-diABZI and nPD-L1-diABZI (Fig. 5a–d). All nanobody carriers were administered I.V. at 1.25 μg of agonist. Additionally, mice were treated with commercially available anti-PD-L1 immune checkpoint blockade (ICB) IgG antibody to model an FDA-approved anti-PD-L1 ICI (e.g., Atezolizumab). A standard preclinical dose of 100 μg ICI was delivered intraperitoneally, which is a near equivalent molar dose of administered nanobody based on antigen binding domains (i.e., single domain for nanobody and two domains for antibody). Remarkably, treatment with AP-diABZI completely eliminated observable EMT6 tumors, resulting in a 100% complete response (CR) rate (10/10 mice) whereas treatment with nAlb-diABZI, while still very effective, yielded a 30% CR rate (3/10 mice); nPD-L1-diABZI only modestly inhibited tumor growth, though to slightly greater extent than the conventional anti-PD-L1 IgG ICB, which conferred only minimal activity in this model. Importantly, no additional toxicity was observed for AP-diABZI relative to nAlb-diABZI (**Extended Data** Fig. 2). To further assess this, we compared AP-diABZI to a combination regimen of nAlb-diABZI and ICB (i.e., anti-PD-L1 IgG) and observed comparably effective antitumor responses, suggesting that the improved efficacy of AP-diABZI over nAlb-diABZI can largely be attributed to immune checkpoint inhibition. Mice treated with AP-diABZI and nAlb-diABZI + ICB that exhibited complete responses were rechallenged 80 days after the initial tumor inoculation with the injection of EMT6 cells in a distal MFP and tumor growth monitored without additional treatment. In both groups, mice were largely resistant to tumor re-challenge with only 1/9 (AP-diABZI) or 1/8 (nAlb-diABZI + ICB) mice developing a tumor with the others remaining cancer free until at least day 100, demonstrating induction of memory lymphocytes that recognize EMT6 tumor antigens (Fig. 5e,f). We next evaluated the antitumor efficacy of AP-diABZI in mice inoculated with parental or PD-L1 knockout EMT6 cells and found that it was less effective (100% vs. 60% CR rate) when PD-L1 was not expressed by breast cancer cells (Fig. 5g,h, **Fig. S24**), potentially due to decreased tumor accumulation and/or reduced checkpoint inhibition. We also evaluated AP-diABZI in a MMTV-PyMT model of spontaneous breast cancer, finding that systemic administration of AP-diABZI significantly reduced tumor burden without evidence of increased lung metastasis (**Extended Data** Fig. 4), which has been implicated as a potentially deleterious consequence of STING signaling in some preclinical models.^59, 60^

To gain insight into the mechanism underlying the increased efficacy of AP-diABZI, we treated mice bearing orthotopic EMT6 tumors with AP-diABZI, nAlb-diABZI, or PBS, collected serum at 4h following the first dose for analysis of serum cytokines (**Fig. S25**), and euthanized mice 24 h after the third dose for gene expression analysis of tumor tissue using the NanoString PanCancer IO 360^™^ panel (**Fig. i-m, Extended Data** Fig. 5). Administration of nAlb-diABZI and AP-diABZI increased serum levels of antitumor IFN-I (IFN-α, IFN-β) and Th1 cytokines (e.g., IL-12, TNF-α) whereas nPD-L1-diABZI did not stimulate response, consistent with its low therapeutic efficacy; interestingly, only AP-diABZI notably increased levels of IFN-γ, a cytokine with an established role in antitumor immunity. Likewise, both nAlb-diABZI and AP-diABZI mediated considerable shifts in the gene expression profile, with transcript signatures associated with increased immune cell infiltrate (immune cell trafficking, CD8^+^ T cells, NK cells, Th1 cells), tumor immunogenicity (antigen presentation, T cell priming, T cell recognition, costimulation, cytokine/interferon signaling), and cancer cell death/apoptosis, with AP-diABZI tending to exert a stronger effect relative to nAlb-diABZI (Fig. 5k–m, **Fig. S26**).

**Figure 6:**
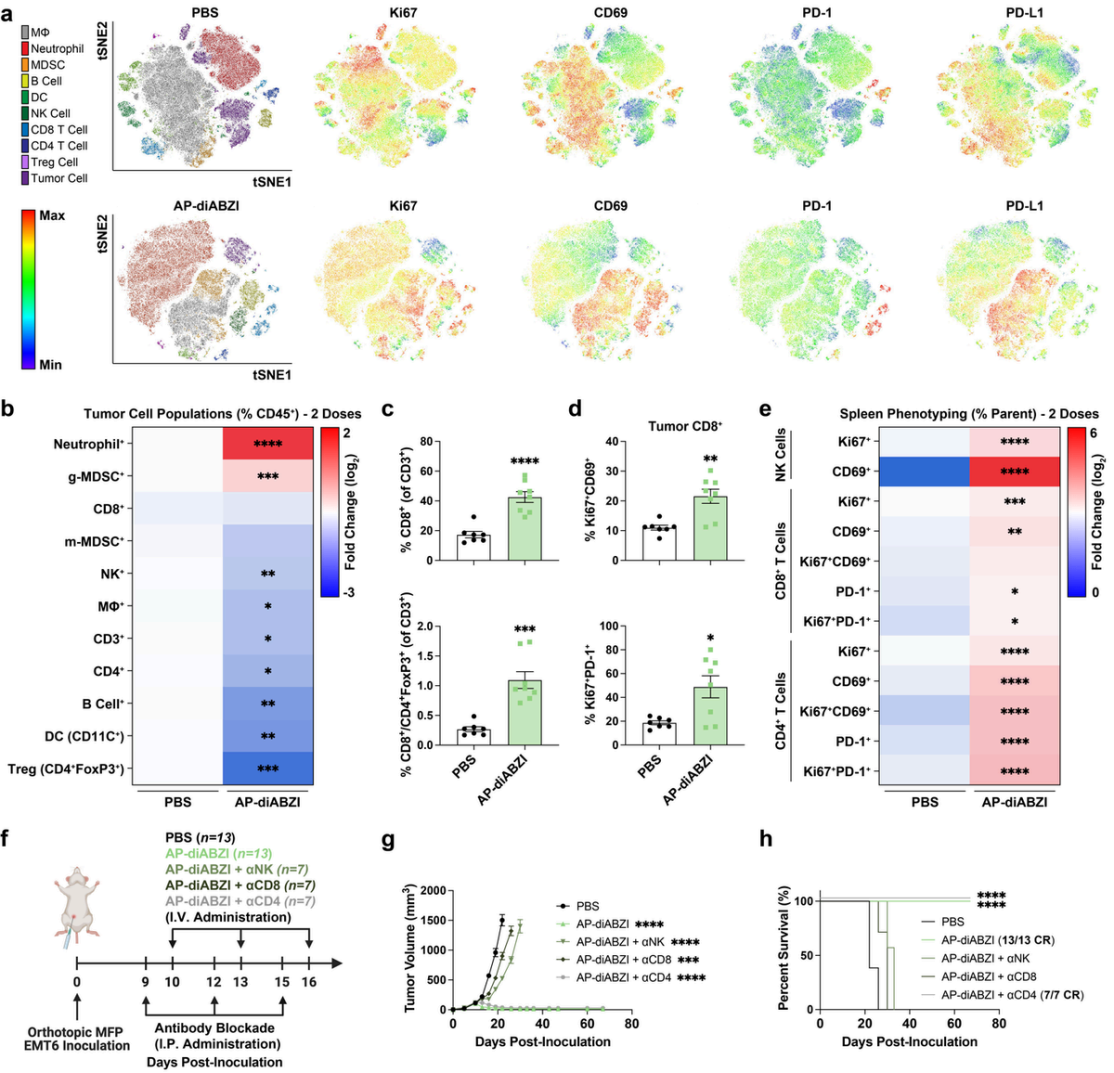
AP-diABZI activates a tumoricidal NK and T cell response. Flow cytometric analysis of orthotopic EMT6 breast tumors 24 h following two intravenous doses of AP-diABZI (1.25 μg, n=8), or PBS (n=7). **(a)** tSNE plots of live cells in EMT6 tumors colored by cell population with relative expression level of Ki67, CD69, PD-1, and PD-L1 as indicated on heat map. DC: dendritic cell; Mj: macrophage; NK: natural killer cell; MDSC: myeloid-derived suppressor cell. **(b)** Heat map summarizing the fold change in the percentage of indicated cell populations in EMT6 tumors. **(c)** Bar plots showing an increase in CD8^+^ cells and the ratio of CD8^+^ to CD4^+^FoxP3^+^ cells (as precent of CD3+ tumor cells). **(d)** Quantification of Ki67^+^CD69^+^ and Ki67^+^PD1^+^ CD8^+^ T cells in EMT6 tumors. *P≤0.05, **P≤0.01, ***P≤0.001, and ****P<0.0001 indicate a statistically significant difference between PBS and AP-diABZI treated groups as determined by Student’s t-test. **(e)** Spleen phenotyping heat map of frequency of NK cells, CD8^+^ T cells, and CD4^+^ T cells. *P≤0.05, **P≤0.01, ***P≤0.001, and ****P<0.0001 indicate a statistically significant difference for heat-maps between PBS and AP-diABZI treated groups as determined by two-way ANOVA. **(f)** Schematic of EMT6 tumor inoculation and treatment schedule with depletion antibodies (n=7–13). Anti-Asialo GM1 (NK) IgG, anti-CD8 IgG, and anti-CD4 IgG were injected I.P. at 100–200 μg and AP-diABZI was injected I.V. at 1.25 μg of diABZI per injection. **(g)** Tumor growth curves, and **(h**) Kaplan-Meier survival plots for mice with EMT6 tumors treated as indicated. CR = complete responder; SEM with *P* value determined by two-way ANOVA with post-hoc Tukey’s correction for multiple comparisons; ****P<0.0001 on day 22 for all groups compared to PBS. Endpoint criteria of 1500 mm^3^ tumor volume with *P* value was determined by log-rank test; ****P<0.0001 compared to PBS control. Replicates are noted as biological, and data shown as mean ± SEM.

To further understand how AP exerts potent antitumor effects, we performed flow cytometric immunophenotyping of EMT6 tumors 48 h following the first dose of nAlb-diABZI and AP-diABZI (**Extended Data** Fig. 6). We observed a decreasing frequency of live cancer cells (CD45^−^) within the tumor and found a significant (*P* < 0.0001) decrease in proliferating (Ki67^+^) cancer cells, consistent with the potent antitumor effects induced by AP-diABZI as well as gene expression analysis supporting increased cancer cell death. Interestingly, there was also an observed trend towards a decrease in PD-L1 expression within cancer cells. We found that a single dose of either nAlb-diABZI or AP-diABZI increased the infiltration of neutrophils and NK cells; more granulocytic MDSCs were also present, potentially contributing as an immunoregulatory mechanism to acute STING activation. However, as observed with nAlb-diABZI treatment, inhibition of MDSCs using SX-682 or anti-GR1 antibody depletion reduced AP-diABZI treatment efficacy (**Fig. S18, S19, S27**). While no change in the overall frequency of CD8^+^ T cells was observed at this early time point, tumor infiltrating CD8^+^ T cells tended to display a more activated phenotype (i.e., CD69^+^), which was also reflected in the splenic T cell population (**Fig. S28** Motivated by these data, we studied the tumor and spleen immune cell dynamics after treatment with one, two, or three doses of AP-diABZI (Fig. 6, **Fig. S29–30**). We found that AP-diABZI increased the frequency of CD4^+^ T cells, CD8^+^ T cells and NK cells expressing markers of activation and proliferation, with a trend towards a stronger response after two and three doses, where a robust antitumor effect was observed (Fig. 6a–e). Consistent with observations following a single dose and the potent anti-tumor efficacy of AP-diABZI, the frequency of CD45^−^Ki67^+^ cancer cells was also reduced (Fig. 6a–d, **Fig. S31**). This is also consistent with gene expression profiling (Fig. 5j–l) indicating increased NK and T cell infiltration and tumoricidal activity. Within the tumor infiltrating T cell compartment, the percentage of CD8^+^ T cells increased with similar trends towards a more activated phenotype, and importantly, the ratio of CD8^+^ cells to FoxP3^+^ regulatory T cells was increased (Fig. 6b,c), indicative of a more immunogenic “hot” immune profile within the TME. Further, within both CD8^+^ and CD4^+^ T cells – both within the tumor and spleen – we observed a shift towards Ki67^+^CD69^+^ and Ki67^+^PD-1^+^ cells, indicating the prevalence of both proliferating and activated lymphocytes in response to AP-diABZI (Fig. 6d,e, **Extended Data** Fig. 7). Together, these data demonstrate that AP-diABZI increases the infiltration of CD8^+^ T cells and NK cells with an activated phenotype and that this effect is enhanced over the use of nAlb-diABZI alone, potentially implicating CD8^+^ T cells and NK cells as the primary antitumor effectors. To test this, we antibody depleted NK cells, CD8^+^ T cells, and CD4^+^ T cells and evaluated antitumor responses elicited by AP-diABZI treatment. Again, we observed a 100% CR rate for AP-diABZI, but treatment efficacy was almost completely inhibited with CD8^+^ T cell NK cell depletion, with CD8^+^ T cell depletion having a slightly stronger effect (Fig. 6f–h); no effect of CD4^+^ T cell depletion was observed. Therefore, both NK cells and CD8^+^ T cells are essential to the potent efficacy of AP-diABZI in an EMT-6 breast tumor model.

### AP-diABZI inhibits B16.F10 tumor growth and primes an antigen-specific memory CD8^+^ T cell response in situ

**Figure 7:**
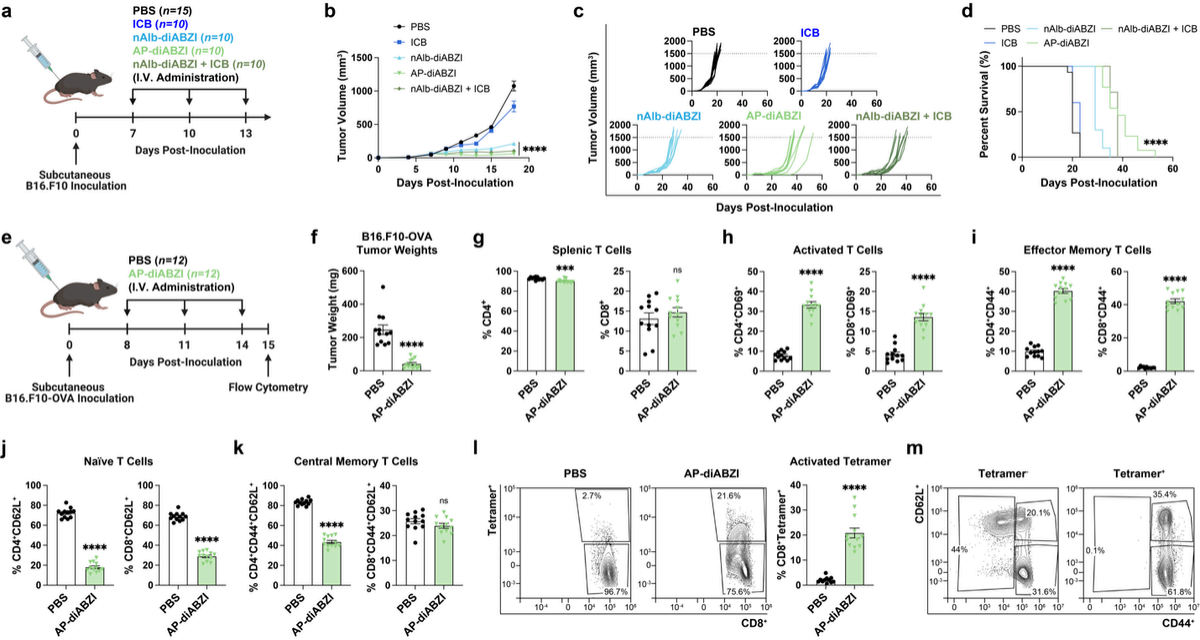
Albumin-hitchhiking STING agonists stimulate antitumor immunity in B16.F10 melanoma tumor model. **(a)** Schematic of B16.F10 tumor inoculation and treatment schedule. **(b)** Tumor growth curves, **(c)** spider plots of individual tumor growth curves, and **(d)** Kaplan-Meier survival plots (n=10–15). Anti-PD-L1 IgG (ICB) was injected I.P. at 100 μg and all nanobodies were injected I.V. at 1.25 μg of diABZI per injection. (b) SEM with *P* value determined by two-way ANOVA with post-hoc Tukey’s correction for multiple comparisons; ****P<0.0001 on day 18 for all groups compared to PBS. (d) Kaplan-Meier survival curves of mice treated with indicated formulation using 1500 mm^3^ tumor volume as endpoint criteria with *P* value was determined by log-rank test; ****P<0.0001 compared to PBS control. **(e)** Schematic of B16.F10-OVA tumor inoculation, treatment schedule, and study end point for flow cytometry analysis (n=12). AP-diABZI was injected I.V. at 1.25 μg of diABZI per injection. **(f)** Tumor weight on day 15 for mice with B16.F10-OVA tumors treated with AP-diABZI or PBS. **(g)** Frequency of CD4^+^ and CD8^+^ T cells in the spleen at study endpoint. Flow cytometric analysis of the frequency of **(h)** CD69^+^ activated T cells, **(i)** CD44^+^CD62L^−^ effector memory T cells, (j) CD44^−^CD62L^+^ naïve T cells, and **(k)** CD44^+^CD62L^+^ central memory T cells. **(l)** SIINFEKL/H-2kB tetramer staining was performed to determine the frequency of OVA-specific CD8^+^ T cells in the spleen at study endpoint. **(m)** Representative flow cytometry dot plots demonstrating the distribution of CD8^+^ T_EM_ (CD44^+^CD62L^−^) and TCM (CD44^+^CD62L^+^) within the OVA-specific (tetramer^+^) and non-OVA-specific (tetramer^−^) populations. *P≤0.05, **P≤0.01, ***P≤0.001, and ****P<0.0001 indicate a statistically significant difference between PBS and AP-diABZI treated groups as determined by Student’s t-test. Replicates are noted as biological, and data shown as mean ± SEM.

We next assessed the efficacy of AP-diABZI in a more challenging and immunosuppressive B16.F10 melanoma model, initiating the three-dose treatment regimen when subcutaneous tumors reached an average size of ~ 75 mm^3^. As expected in this model, anti-PD-L1 ICB exerted no therapeutic benefit, whereas both nAlb-diABZI and AP-diABZI suppressed tumor growth and elongated median survival, with AP-diABZI conferring the most survival benefit, consistent with findings in the EMT6 model (Fig. 7a–d). We also found that AP-diABZI was more effective than free diABZI administered at 24 times (30 μg) the dose in the B16.F10 model (**Fig. S32**). We again evaluated cytokine levels in plasma 4 h following the first injection using a multiplexed ELISA and found that anti-PD-L1 ICB increased only IL-1α levels, while nAlb-diABZI and AP-diABZI stimulated the production of cytokines associated with antitumor immunity, including IFN-α, IFN-β, IFN-γ, IL12p70, and CXCL10 (**Extended Data** Fig. 8). To determine the primary cellular effectors to AP-diABZI in the B16.F10 model, we antibody depleted CD4^+^ and CD8^+^ T cells and NK cells again finding that the antitumor response was mediated predominantly by CD8^+^ T and NK cells (**Extended Data Fig. 9**)

STING activation can prime the immune system to stimulate a systemic, antigen-specific, antitumor T cell responses with potential to lead to generation of T cell memory.^19, 61^ Given evidence of increased antigen presentation, cancer cell killing, and T cell priming (Fig. 5), as well as protection from tumor re-challenge in mice with EMT6 tumors treated with AP-diABZI, we next assessed the capacity of AP-diABZI to stimulate a *de novo* tumor antigen-specific CD8^+^ T cell response. To test this, we inoculated C57BL/6 mice with B16.F10 melanoma cells expressing ovalbumin (B16.F10-OVA) as a model antigen and treated mice with either PBS or AP-diABZI on a three-dose regimen once tumors reached a size of 75–100 mm^3^ (Fig. 7e–m). 24 h after the final dose, mice were euthanized for flow cytometric evaluation of splenic T cell response. Consistent with results in mice with parental B16.F10 tumors, AP-diABZI treatment significantly (*P* < 0.0001) reduced tumor burden (Fig. 7f). Treatment with AP-diABZI resulted in a significant (*P* < 0.0001) increase in activated CD69^+^ CD4^+^ and CD8^+^ T cells (Fig. 7h) and effector memory (CD44^+^CD62L^−^) CD4^+^ and CD8^+^ T cells, with a reduction in CD4^+^ central memory (CD44^+^CD62L^+^) T cells. Using SIINFEKL/H-2Kb tetramer staining, we also found that AP-diABZI treatment stimulated a strong peripheral OVA-specific CD8^+^ T cell response (Fig. 7l), characterized by a predominantly (~ 60%) CD44^+^CD62L^−^ effector memory phenotype (Fig. 7m, **Extended Data Fig. 10**). Hence, in addition to remodeling the TME, systemic administration of AP-diABZI primes antigen-specific CD8^+^ T cell effector and memory responses capable of targeting tumor-associated antigens.

### Albumin-hitchhiking STING agonists inhibit lung metastatic disease

**Figure 8:**
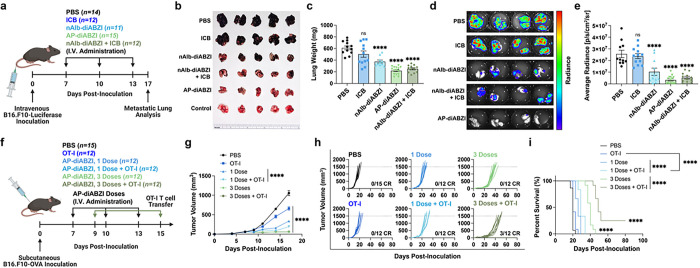
Albumin-hitchhiking STING agonists improve immunotherapy responses in a model of lung metastatic melanoma and adoptive T cell transfer therapy. **(a)** Schematic of B16.F10-LUC I.V. tumor inoculation, treatment schedule, and study end point for analysis of lung tumor burden (n=11–15). Anti-PD-L1 IgG (ICB) was injected I.P. at 100 μg and all nanobodies were injected I.V. at 1.25 μg of diABZI per injection. **(b)** Representative images of lungs and **(c)** lung weights of mice treated as indicated. **(d)** Representative IVIS luminescent images and **(e)** quantification of average radiance from luciferase expressing B16.F10 within isolated lung tissue. *P* values determined by one-way ANOVA with post-hoc Tukey’s correction for multiple comparisons; *P≤0.05, **P≤0.01, ***P≤0.001, and ****P<0.0001 compared to the PBS control. **(f-i)** Evaluation of AP-diABZI as an adjuvant therapy for adoptive OT-I T cell transfer therapy in a B16.F10-OVA model (n=15). **(f)** Schematic of B16.F10-OVA tumor inoculation and of treatment schedule with OT-I transfer (0.5 million OT-I T cells) either on day 9 (OT-I alone or one dose (1.25 μg) AP-diABZI pre-treatment) or day 15 (three dose AP-diABZI pre-treatment). **(g)** Tumor growth curves, **(h)** spider plots of individual tumor growth curves, and **(i)** Kaplan-Meier survival curves. (g) *P* value determined by two-way ANOVA with post-hoc Tukey’s correction for multiple comparisons; ****P<0.0001 on day 17 for all groups compared to PBS. (i) Kaplan-Meier survival curves of mice treated with indicated formulation using 1500 mm^3^ tumor volume as endpoint criteria with *P* value was determined by log-rank test; ****P<0.0001 compared to PBS control. (CR = complete responder). Replicates are noted as biological, and data shown as mean ± SEM.

Based on the evidence that AP-diABZI can stimulate an effective antitumor immune response in the immunologically “cold” B16.F10 model, we extended our investigations to evaluate therapeutic efficacy in an aggressive model of lung metastatic melanoma induced through intravenous inoculation of luciferase-expressing B16.F10 (B16.F10-Luc) cells (Fig. 8a). A week following inoculation, we used the three-dose combination therapy regimen described previously. On day 17 post-inoculation, mice were euthanized and lungs were harvested for quantification of tumor burden via measurement of lung mass, immunohistochemistry, and bioluminescent imaging (Fig. 8b–e, **Fig. S33**). High metastatic tumor burden was evident in mice receiving anti-PD-L1 ICB alone, but significantly (*P* < 0.0001) reduced in mice receiving nAlb-diABZI and nearly eliminated in mice receiving AP-diABZI. Importantly, these data show that albumin-hitchhiking STING agonists are effective against metastases in the lung, one of the most common metastatic sites for many cancers. This also suggests a potential to treat micrometastases, which typically lack the leaky vasculature required for tumor accumulation via the enhanced permeation and retention effect;^62^ by contrast, albumin-binding molecules have been shown to accumulate in micrometases.^63^

### AP-diABZI opens a therapeutic window for adoptive T cell transfer therapy

Finally, we sought to demonstrate the versatility of the platform by extending the application of AP-diABZI to the setting of adoptive cellular immunotherapy,^64^ which includes tumor infiltrating lymphocyte (TIL) therapy, CAR T cells, and TCR-engineered T cells that face major barriers to tumor infiltration and function, which continues to limit their clinical impact in the treatment of solid tumors.^65, 66^ Founded on data demonstrating that nAlb-diABZI and AP-diABZI enhance the infiltration of endogenous antitumor T cells, we hypothesized that the approach could be used to pre-condition the TME to generate a therapeutic window for adoptive T cell therapy. To test this, we inoculated female C57BL/6 mice with subcutaneous B16.F10-OVA cells and allowed the tumors to reach approximately 75 mm^3^ (Fig. 8f). We then treated mice with either one or three doses of AP-diABZI, followed by a single intravenous dose of activated OVA-specific activated CD8^+^ T cells (OT-I T cells). Treatment with OT-I cells only (no STING agonist) on day 9 resulted in marginal therapeutic benefit (Fig. 8g–h), consistent with the highly immunosuppressive B16.F10 TME that restricts T cell infiltration and effector function. However, treatment with OT-I T cells 48 h after either one or three AP-diABZI doses conferred significant (*P* < 0.0001) reduction in tumor growth and prolonged mouse survival (Fig. 8i). Importantly, the treatment regimen of three doses of AP-diABZI prior to one dose of OT-I T cells resulted in a 25% complete response rate (3/12 mice). This provides additional evidence that albumin-hitchhiking STING agonists can establish an inflammatory milieu that supports T cell infiltration and function. While here we employed a simplified model of an adoptive T cell therapy, these studies highlight the potential to leverage nanobody-STING agonist conjugates to enhance responses to multiple T cell-based immunotherapies, including autologous TIL therapy, CAR T cells, and cancer vaccines.

## Discussion

Innate immunity fuels the cancer immunity cycle, playing critical roles in antitumor T cell priming, recruitment of cytotoxic immune cells, and recognition of tumor antigens.^67–69^ However, the development of innate immune agonists targeting specific PRRs has been limited by pharmacological barriers that have largely restricted their application to intralesional administration,^4^ which has yet to deliver on its clinical promise.^11^ This challenge has been recently exemplified by the clinical exploration of STING agonists, which have demonstrated impressive results when administered intratumorally in mouse models but have not yet proven effective in patients. To address this, we developed a platform for systemic delivery of STING agonists based on an albumin hitchhiking nanobody (nAlb) engineered for precisely defined and site-selective ligation of a DBCO-functionalized “clickable” diABZI cargo that we synthesized. Our data demonstrates that intravenously administered nAlb conjugates bind to circulating albumin *in situ*, increasing nanobody half-life from minutes to days and harnessing the capacity of albumin to accumulate in tumors to delivery of cargo to cancer cells and tumor-associated myeloid cells in the TME. This triggered potent STING activation at tumor sites, initiating an inflammatory program that increased the infiltration of activated NK cells and CD8^+^ T cells with antitumor function. Accordingly, nAlb-diABZI conjugates exhibit improved efficacy in mouse models of breast cancer and melanoma relative to a leading free diABZI agonist.

An appealing feature of anti-albumin nanobodies, and other protein-based albumin hitchhiking agents (e.g., affibodies, humabodies) over other albumin binders (e.g., lipids, Evans blue) is the high degree of molecular programmability that can be achieved through protein engineering. Here we illustrate this modularity by recombinantly integrating a PD-L1 binding domain to create a bi-valent fusion protein for covalent conjugation of diABZI. This yielded a single, well-defined, multifunctional STING agonist that increased tumor accumulation in a PD-L1-dependent manner, while also blocking an important immune checkpoint, resulting in spontaneous induction of tumor antigen-specific T cells that inhibited tumor growth and provided immunological memory that protected against tumor rechallenge. While we selected PD-L1 on translational considerations, the bivalent nanobody platform is readily amenable to integration of other immunoregulatory features and/or molecular targeting ligands. To date, there are sparingly few reports describing the targeted delivery of STING agonists,^70, 71^ with most employing surface-decorated nanoparticles for CDN delivery.^72–74^ Our albumin hitchhiking nanobody approach offers several potential advantages including plug-and-play programmability, precise and site-selective ligation of STING agonists, and a smaller size that has been reported to increase improve tumor penetration, a limitation of nanoparticles and full-length antibodies in tumors with dense stroma.^75–77^

Though there are vast future opportunities for bivalent nanobody-agonist conjugates, it is also notable that nAlb-diABZI was highly effective as a single agent, which may be advantageous for cancers that lack a defined cell surface target (e.g., triple negative breast cancer, melanoma). However, clinical imaging has demonstrated that albumin accumulation varies amongst cancer types and patients^29^ and the implications of this for the activity and efficacy of nAlb-diABZI must be considered and further investigated. To date, most research on albumin-based drug carriers for cancer (e.g., Abraxane, aldoxorubicin) has focused on delivery of chemotherapy drugs that target cancer cells. By contrast, immunostimulatory agents such as STING agonists can stimulate complex antitumor immunological programs that may be more dependent on immunological variables (e.g., neoantigen load, immune status of the TME) than on the efficiency of drug accumulation in tumor tissue or delivery to cancer cells. For example, in our analysis of nAlb-Cy5 biodistribution, we found ~ 11% ID/gram tumor in the EMT6 model and a comparable ~ 8.4% ID/gram tumor in B16.10 model (Fig. 2), yet a substantial difference in response to both nAlb-diABZI alone and in combination with anti-PD-L1 that may be attributed to the relatively low immunogenicity to B16.F10 tumors. Importantly, the efficacy of nAlb-diABZI was enhanced when delivered in combination with anti-PD-L1 ICB and therefore may hold promise when combined with other ICIs and as an adjuvant therapy for patients with acquired resistance to ICIs. Additionally, nAlb-diABZI was much more effective than nPD-L1-diABZI, which was cleared rapidly with minimal tumor accumulation despite a capacity to activate STING, bind PD-L1, and inhibit immunoregulatory PD-L1/PD-1 signaling.^78^ This finding contributes to an evolving understanding of how the spatiotemporal dynamics of immunomodulatory signals impact the efficacy and safety of systemically delivered innate immune agonists and other immunotherapies.^54, 79–81^ Indeed, anti-albumin nanobodies have been engineered with variable affinity^82^ and this may afford a future opportunity for precisely modulating plasma half-life to establish immunopharmacological principles to optimize systemic innate immune agonist delivery.

Also critical to the efficacy of our technology was the design and synthesis of a diABZI STING agonist functionalized with a DBCO group for biorthogonal conjugation to azide-presenting nanobodies. Despite being stably linked to the nanobody via an amide bond, the STING agonist exhibited high potency *in vitro* and *in vivo*, which we attribute to lysosomal degradation of endocytosed diABZI-nanobody conjugates and release of an active species (**Fig. S6, Extended Data** Fig. 1). While there may be an advantage to using such stable linkers to minimize premature drug release into the circulation^83^, our strategy also opens the possibility of installing cleavable linkers (e.g., enzyme cleavable, ROS cleavable) that enable environmentally responsive, “logic-gated” drug release with potential to further improve tumor specificity and reduce systemic exposure.^84, 85^ Additionally, while our selection of a diABZI agonist was largely motivated by their recent advancement into clinical trials, the platform is also amenable to conjugation to other STING agonists (e.g., recently described conjugatable CDNs)^18, 22^ as well as agonists targeting other PRRs.^86, 87^

In summary, we have integrated synthetic biology tools to engineer precisely defined nanobody-STING agonist conjugates as a platform for cancer immunotherapy. We leveraged albumin binding nanobodies as a scaffold from which diverse immunomodulatory components can be readily integrated via recombinant and chemical design. We demonstrated that albumin-hitchhiking nanobodies enhanced the potency and efficacy of a diABZI STING agonist and we showcased the versatility of the system by introducing an PD-L1 binding nanobody that affords increased tumor targeting and immune checkpoint inhibition to further potentiate antitumor immunity and efficacy. We found nanobody-diABZI conjugates to be highly effective in an orthotopic breast cancer model and an aggressive model of lung metastatic melanoma and we further demonstrated their utility an adjuvant therapy to improve responses to adoptive T cell transfer. Importantly, nanobody-diABZI conjugates were well-tolerated with a favorable preclinical toxicity profile and are amenable to established scalable manufacturing workflows and translational pipelines. Collectively, our study establishes a preclinical foundation for future development of nanobody- and other protein-STING agonist conjugates as an enabling platform for cancer immunotherapy.

## Methods

### Cell Lines and Materials.

All chemicals involved in synthesis of target compounds were reagent grade unless stated otherwise. DNase, isopropyl thiogalactoside (IPTG), and dimethyl sulfoxide (DMSO) were purchased from Sigma-Aldrich. Azido-PEG_3_-Amine and DBCO-PEG_12_-NHS Ester were purchased from Broadpharm. Magnesium sulfate, sodium hydroxide, sodium azide, sodium acetate, sodium azide, sodium chloride, sodium bicarbonate, sodium hydroxide, 2xYT media, kanamycin, protease inhibitor cocktail tablets (EDTA free), Nickel NTA resin, and all other organic solvents were purchased from Thermo Fisher Scientific. All DNA block segments involved in cloning protein inserts were purchased from Integrated DNA Technologies (IDT) with standard desalting as means of purification. For protein expression, pET28-b(+) expression vector, Q5 Hot Start Master Mix 2x, T4 DNA ligase, Golden Gate Master Mix (BsaI-HF v2), DH5α *E. coli*, and T7 SHuffle Express *E. coli* chemically competent cells were purchased from New England Biolabs (NEB). Qiaprep Miniprep Spin kits were purchased from Qiagen. THP1-Dual and A549-Dual cell lines were purchased from InvivoGen. A549-Dual cells were cultured in Dulbecco’s Modified Eagle Medium (DMEM; Gibco) and was supplemented with 2 mM L-glutamine, 4.5 g/L glucose, 10% heat-inactivated fetal bovine serum (HI-FBS; Gibco), 100 U ml − 1 penicillin/100 μg ml − 1 streptomycin (Gibco), and 100 μg/mL Normocin. THP1-Dual cells were cultured in Roswell Park Memorial Institute (RPMI) 1640 Medium (Gibco) and was supplemented with 2 mM L-glutamine, 4.5 g/L glucose, 10% heat-inactivated fetal bovine serum (HI-FBS; Gibco), 100 U ml − 1 penicillin, 100 μg ml − 1 streptomycin (Gibco), and 100 μg/mL Normocin. Every other passage, both Blasticidin (InvivoGen) and Zeocin (InvivoGen) were added at a concentration of 200 μg/mL to the cell culture flask. The murine breast cancer cell line EMT6 and melanoma cell lines B16.F10 and B16.F10-LUC2 were purchased from American Type Culture Collection (ATCC), where EMT6 cells were grown in RPMI supplemented with 2 mM L-glutamine, 4.5 g/L glucose, 10% HI-FBS, and 100 U ml − 1 penicillin and 100 μg ml − 1 streptomycin. B16.F10 and B16.F10-LUC2 cells were cultured in DMEM supplemented with 2 mM L-glutamine, 4.5 g/L glucose, 10% heat-inactivated fetal bovine serum, and 100 U ml − 1 penicillin and 100 μg ml − 1 streptomycin. B16.F10-OVA cells were a gift from Dr. Amanda Lund and were cultured in DMEM supplemented with 2 mM L-glutamine, 4.5 g/L glucose, 10% heat-inactivated fetal bovine serum, and 100 U ml − 1 penicillin and 100 μg ml − 1 streptomycin with continuous selection using Geneticin (G418; Gibco) after every cell passage at a concentration of 500 μg/mL. All cell types used in the study were grown in a humidified atmosphere at 37°C in 5% CO2.

### Cloning of Proteins.

Gene cassette was purchased from IDT in the form of a gene block, with cloning restriction sites placed on both flanking regions (BsaI – GGTCTC). In the case of a fusion protein, a genetic sequence was placed between the two domains (XTEN – SGSETPGTSESA). For sortase mediated bioconjugation of nanobodies, a C-terminal sequence was incorporated (LPETGGHHHHHHEPEA). The gene fragment was digested with BsaI-HF v2 in a golden gate master mix (New England Biolabs) and ligated into a pET28-b(+) plasmid. We transformed the construct into chemically competent DH5 α (New England Biolabs) *E. coli* and plated on LB agar with Kanamycin. We transformed the sequence-verified pET28b plasmid into T7 Shuffle Express (New England Biolabs) *E. coli* as the expression strain. Glycerol stocks were maintained at −80 °C of every successfully transformed bacterial strain.

### Engineered Sortase A (eSrtA) Sequence (No Start Codon).

#### eSrtA – HisTag

QAKPQIPKDKSKVAGYIEIPDADIKEPVYPGPATREQLNRGVSFAEENESLDDQNISIAGHTFIDRPNYQFTNLKAAKKGSMVYFKVGNETRKYKMTSIRNVKPTAVEVLDEQKGKDKQLTLITCDDYNEETGVWETRKIFVATEVKLEHHHHHH

Formula: C_785_H_1234_N_220_O_242_S_3_

Molecular Weight: 17721.94 Da

〿_280_ = 14440 M-1 cm-1

### Anti-Albumin (nAlb) Sequence (No Start Codon).

#### nAlb – Ligation Tag

EVQLVESGGGLVQPGGSLRLSCAASGFTFRSFGMSWVRQAPGKEPEWVSSISGSGSDTLYADSVKGRFTISRDNAKTTLYLQMNSLKPEDTAVYYCTIGGSLSRSSQGTQVTVSSLPETGGHH HHHHEPEA

Formula: C_606_H_939_N_175_O_198_S_4_

Molecular Weight: 13972.42 Da

〿_280_ = 17085 M-1 cm-1

### Anti-EGFR (nEGFR) Sequence (No Start Codon).

#### nEGFR – Ligation Tag

QVKLEESGGGSVQTGGSLRLTCAASGRTSRSYGMGWFRQAPGKEREFVSGISWRGDSTGYADSVKGRFTISRDNAKNTVDLQMNSLKPEDTAIYYCAAAAGSAWYGTLYEYDYWGQGTQVTVSSLPETGGHHHHHHEPEA

Formula: C_662_H_996_N_194_O_214_S_4_

Molecular Weight: 15224.60 Da

〿_280_ = 34045 M-1 cm-1

### Anti-GFP (nGFP) Sequence (No Start Codon).

#### GFP – Ligation Tag

QVQLQESGGALVQPGGSLRLSCAASGFPVNRYSMRWYRQAPGKEREWVAGMSSAGDRSSYEDSVKGRFTISRDDARNTVYLQMNSLKPEDTAVYYCNVNVGFEYWGQGTQVTVSSLPETGGHH HHHHEPEA

Formula: C_631_H_955_N_189_O_200_S_5_

Molecular Weight: 14548.97 Da

〿_280_ = 27055 M-1 cm-1

### Anti-PD-L1 (nPD-L1) Sequence (No Start Codon).

#### nPD-L1 – Ligation Tag

QVQLQESGGGLVHPGGSLRLSCATSGSIFSIISMGWYRQAPGKQRELVALVFRGGSTVYADSVKGRFTISGDIAKSTVYLQMDSLKPEDTAVYYCNAKPIGTAQYWGQGTQVTVSSLPETGGHH HHHHEPEA

Formula: C_625_H_966_N_178_O_192_S_4_

Molecular Weight: 14173.86 Da

〿_280_ = 20065 M-1 cm-1

### Anti-Albumin – Anti-PD-L1 (AP) Sequence (No Start Codon).

#### nAlb – XTEN Linker – nPD-L1 – Ligation Tag

EVQLVESGGGLVQPGGSLRLSCAASGFTFRSFGMSWVRQAPGKEPEWVSSISGSGSDTLYADSVKGRFTISRDNAKTTLYLQMNSLKPEDTAVYYCTIGGSLSRSSQGTQVTVSSSGSETPGTSESAQVQLQESGGGLVHPGGSLRLSCATSGSIFSIISMGWYRQAPGKQRELVALVFRGGSTVYADSVKGRFTISGDIAKSTVYLQMDSLKPEDTAVYYCNAKPIGTAQYWGQGTQVTVSSLPETGGHHHHHHEPEA

Formula: C_1195_H_1863_N_337_O_388_S_8_

Molecular Weight: 27415.44 Da

〿_280_ = 37150 M-1 cm-1

### Expression and Purification of Proteins.

5 μL of Kanamycin (stocked at 50 mg/mL) was added to a culture tube containing 5 mL 2xYT media and inoculated with a stab of protein (cloned into a NEB T7 Shuffle Express cell line). The culture was incubated at 30 °C, with shaking at 250 RPM, for 16 hours. Each culture was transferred to a 2 L baffled flask containing 500 mL of autoclaved 2xYT media and 500 μL of Kanamycin (25 mg) and shaken at 30 °C in an Innova 42R (New Brunswick Scientific) incubator for 4.5–5 hours (until the OD_600_ reached ~ 0.8). The cultures were then cooled to ~ 16°C and induced with IPTG (2.5 mM final concentration). The induced cultures were shaken overnight (20–24 hours) at 16 °C. The bacteria were harvested the next day by centrifugation (3900 rpm for 10 min) and the pellet was reconstituted in 1x PBS with Dnase I and a tablet of protease inhibitor cocktail (EDTA free). The cells were lysed by sonication on an ice bath in 5 second increments over 10 minutes. The resulting bacterial lysate was centrifuged (11000 rpm for 20 min) to remove cellular debris. The supernatant was added to a 50 mL Kontes Flex column (Kimbal Kontes Glassware) containing 3 mL of Nickel-NTA histidine binding resin that was preequilibrated with 1x PBS buffer. This column was placed on a rotating shaker at room temperature for 1–2 hrs. After this period, the supernatant was drained from the column using gravity and the column washed with 1x PBS buffer twice. Weakly bound proteins were first washed off the resin using a low concentration elution buffer (2× 10 mL, 10 mM imidazole, 1x PBS pH 7.4 @ 25 °C). The bound protein was then eluted from the resin using elution buffer (15 mL, 150 mM imidazole, 1x PBS pH 7.4 @ 25 °C). The eluate was then concentrated to 0.5 mL in a 15 mL Microcon 10 kDa Centrifugal Filter Unit (Millipore) and subsequently purified by size exclusion chromatography (SEC) via an Akta FPLC (Cytiva), on a Hi-Load 16/60 Superdex 200 column using 1x PBS, pH 7.4 as the running buffer at 4 °C. Pure fractions were determined by SDS-PAGE, pooled together with buffer exchange to 1x PBS, and stocked at either − 20 °C or 4 °C.

### Enzymatic Bioconjugation and Click Chemistry Reactions.

Bioconjugation reactions occurred in mild conditions (20 mM HEPES at pH 7.4, 150 mM NaCl, and 10 mM CaCl_2_) between eSrtA (100 μM) and a nanobody containing a C-terminal ligation tag (75 μM) using a primary amine containing functional group (20 mM). Reactions occurred with mixing by a rotary shaker overnight (16 h) and were quenched by the addition of a 1:1 volume of a chelating agent EDTA containing solution (20 mM HEPES at pH 7.4, 300 mM NaCl, and 10 mM EDTA) under rotation for one hour. After the reaction was stopped, the solution was concentrated and buffer exchanged to 1x PBS (without NaCl or MgCl_2_) three times by centrifugal dialysis. The protein solution was then immobilized to Nickel-NTA histidine binding resin at least 2 hours, and unbound protein was collected by washing the resin with 1x PBS. For nanobodies that contain a histidine in the native sequence, proteins were eluted in mild conditions (10 mM Imidazole in 1x PBS). Collected protein was concentrated and buffer exchanged to 1x PBS by centrifugal dialysis and verified by ESI-MS and SDS-PAGE. Click chemistry reactions proceeded by the addition of 5 eq. (molar) of the complementary handle (e.g. if an azide was placed on the nanobody, the click chemistry reaction would proceed with the addition of 5 eq. of DBCO-containing moiety). For Cy5 conjugations, sulfo-Cy5 was used from Sigma Aldrich, and Cy5 was also used from Broadpharm. After 48 hours of reaction between the protein-azide and the DBCO-moiety under rotation at room temperature, the mixture was purified by centrifugal dialysis four times with 1x PBS, and verified for purity by UV-VIS, ESI-MS, and SDS-PAGE.

### Sodium Dodecyl Sulfate-Polyacrylamide Gel Electrophoresis (SDS-PAGE).

Protein samples were diluted in 1x PBS to 10 μM before analysis. 10 μL of the protein sample was mixed with 10 μL of reducing Laemmli buffer. Samples were boiled at 95 °C for 5 minutes, and 15 μL of each sample was loaded into a 15-well, 4–15% Tris-glycine precast polyacrylamide gel (Biorad) and ran at a constant 150 V with 343 mA for 30 minutes. The gel was then either first imaged for fluorescence on a UV-transilluminator or directly stained using Coomassie-B-250.

### Electrospray Ionization Liquid Chromatography Mass Spectrometry (ESI-MS).

Proteins were buffer exchanged into ammonium acetate (pH 5.5) and concentrated to approximately 100 μM. ESI-MS data were collected using an Agilent 6210A time-of-flight (TOF) mass spectrometer at a range of 50 − 20,000 m/z over a period of two minutes. Data were analyzed with Agilent MassHunter IM-MS Acquisition Data software to reveal m/z data, where files were condensed across the two-minute run. These m/z data were deconvoluted using a maximum entropy deconvolution calculation using UniDec to give the deconvoluted mass spectra using background subtraction between a range of 1,000–5,000 m/z and with an export range of 5,000–50,000 Da.

### Computational Modeling and Analysis of nAlb Nanobody.

nAlb was modeled in silico using RoseTTAFold (GitHub; RosettaCommons) and binding between HSA (PDB: 1AO6) and nAlb was predicted using RosettaDock through ROSIE (Rosetta Online Server that Includes Everyone; Pittsburgh). After an initial screening for best fits of the docking between HSA (receptor) and nAlb (ligand), the best fit model was then returned for rescreening to confirm an optimal energy conformation between the structures. The final structures of nAlb and the bound nAlb-HSA complex were exported to PyMOL for generating a figure of the structure.

### Isothermal Titration Calorimetry.

All proteins used were equilibrated in buffer at indicated pH values by titration using HCl (aq.) or NaOH (aq.) in PBST, 150 mM NaCl, 3 mM EDTA, 0.05% Tween 20. Albumin (HSA and rMSA) and PD-L1 receptor titrations were run on the TA Instruments Affinity ITC instrument (TA Instruments, New Castle, DE). 350 μL of albumin or the PD-L1 receptor was added to the sample cell (10–20 μM), and either the nAlb nanobody (125–250 μM) or nPD-L1 nanobody (250 μM) was loaded into the injection syringe, respectively. The reference cell contained ultrapure water and was changed after each titration experiment. All runs used the following instrument settings: cell temperature 298 K, reference power 10 μCal/second, initial delay 240 seconds, stirring speed 75 rpm, feedback mode/gain high, and injection volume 2 μL for 10 seconds, titration spacings at 120 second intervals, and a filter period of 10 seconds. Data were analyzed using the provided NanoAnalyze software for the instrument to determine thermodynamics of binding from an independent model.

### Tritosome Degradation Assay and MALDI-TOF MS.

Tritosomes (BioIVT) were prepared and activated by combining 70 μL of nuclease-free water, 10x of catabolic buffer (K5200, BioIVT), and 100 μL of pure lysosomes (H0610.L, BioIVT) and incubating the mixture at 37 °C for 15 minutes. Samples for lysosomal degradation were added at 0.5 μM (10 μL) with the tritosome mixture and incubated at 37 °C over a period of 48 hours. Aliquots were taken from the reaction mixture at distinct time points and flash frozen with liquid nitrogen and stored at −80 °C to stop the reaction. Activity was determined by observing molecular weight shifts in the substrate using matrix assisted laser desorption and ionization time-of-flight mass spectrometry (MALDI-TOF MS). 3 μL of matrix (15 mg mL-1 THAP in dry acetone) was combined with 1 μL of aliquoted sample and spotted on a stainless steel MALDI-MS plate (Bruker). Matrix was evaporated using compressed-air, read on a Bruker AutoFlex MALDI-TOF, and processed with the FlexControl software (Bruker Daltonics). The laser pulse rate was 500 Hz and spectra were obtained with a mass window of 400–4000 m/z at the highest resolution for the instrument (4.00 GS/s). FlexAnalysis software (Bruker Daltonics) was used to obtain baseline spectra for all samples. Data were exported and plotted using MATLAB to generate figures showing m/z spectra at distinct time points.

### Synthesis and NMR Verification of DBCO-PEG_11_ -diABZI.

Synthesis of the DBCO conjugated STING agonist (diABZI) is reported in **Fig. S2**, with NMR verification in **Fig. S3–4**. We first generated a STING agonist that features a reactive amine handle, which was synthesized in four steps. Briefly: Aryl amination of an aryl chloride **1** with an amine **2** gave a di-nitro analog, compound **3**. The di-nitro compound **3** was subjected to reduction using sodium dithionite in methanol, generating a di-amine moiety **4**. Compound **4** was then treated with isothiocyanate, followed by EDC coupling, to reveal a boc-protected analog, compound **5**. Next, the boc-group from compound **5** was deprotected by treating with TFA:DCM. To a stirred solution of amine **6** (100 mg, 0.089 mmol, 1 eq.) in 5 mL DMF, hunig’s base was added (77 μL, 0.44 mmol, 5 eq.) under argon atmosphere at room temperature. After stirring for 5 min, a solution of activated NHS ester (98 mg, 0.098 mmol, 1.1 eq.) in DMF (5 mL) was added dropwise and stirred overnight (16 h). The solvent was evaporated to get crude product **7**, which was purified by silica gel column chromatography using a mixture of Methanol/Dichloromethane as an eluent (5–25% MeOH) to get the desired product as a solid (70mg, 0.042mmol, yield 43%). (R_f_ = 0.5 in 20% MeOH in DCM). ^1^H NMR (400 MHz, DMSO) δ 8.01–7.93 (m, 2H), 7.88 (t, *J* = 5.7 Hz, 1H), 7.75 (t, *J* = 5.7 Hz, 1H), 7.67–7.60 (m, 4H), 7.49–7.42 (m, 3H), 7.38–7.27 (m, 7H), 6.49 (d, *J* = 7.1 Hz, 2H), 5.88–5.79 (m, 2H), 5.01 (d, *J* = 13.9 Hz, 1H), 4.91 (dd, *J* = 29.6, 4.2 Hz, 4H), 4.53–4.49 (m, 4H), 3.98 (t, *J* = 6.0 Hz, 2H), 3.72 (s, 3H), 3.60–3.57 (m, 2H), 3.54 (t, *J* = 6.5 Hz, 2H), 3.47 (broads, 46H), 3.30–3.26 (m, 2H), 3.14–3.05 (m, 4H), 2.26 (t, *J* = 6.5 Hz, 2H), 2.09 (s, 3H), 2.08 (s, 3H), 2.01–1.96 (m, 1H), 1.78–1.72 (m, 1H), 1.68 (p, *J* = 6.5 Hz, 2H), 1.28–1.27 (m, 6H). ^13^C NMR (151 MHz, DMSO) δ 171.57, 171.50, 170.52, 168.06, 167.33, 152.50, 152.45, 152.06, 148.88, 145.50, 145.28, 144.65, 140.37, 140.33, 132.87, 130.54, 130.49, 130.07, 129.37, 128.62, 128.58, 128.44, 128.25, 128.13, 127.24, 125.60, 122.99, 121.86, 120.11, 120.04, 114.67, 109.72, 108.61, 106.00, 105.83, 105.58, 70.21, 70.14, 70.10, 70.00, 69.94, 69.45, 67.26, 56.45, 55.34, 53.85, 46.05, 45.05, 42.12, 38.95, 36.57, 35.61, 30.80, 30.17, 29.13, 18.46, 17.17, 16.58, 13.57, 12.74. HRMS (ESMS) Calculated for C_84_H_111_N_15_O_21_ [M + Na]^+^: 1688.7977, found 1688.7982.

### Synthesis and NMR Verification of Amine-PEG_3_ -Triazole-PEG_11_ -diABZI.

To a stirred solution of amine-PEG_3_-azide (3.14 mg, 14.4 μmol, 3 eq) in a 1:1 MeCN:H_2_O mixture (4 mL), hunig’s base (3.10 mg, 24.0 μmol, 5 eq) and 450 μL of a stock solution of 10.8 millimolar DBCO-PEG_11_-diABZI in DMSO (8.0 mg, 4.8 μmol, 1 eq) were added and stirred overnight. Acetonitrile was removed by slowly passing an air stream through the reaction flask. When the reaction mixture reduced to half, the aqueous mixture was frozen at −80 °C for 8 h and then lyophilized. Diethyl ether was added (×3) and vigorously shaken with diethyl ether. The mixture was decanted to remove excess amine-PEG3-azide. After three washes with diethyl ether, it was dried overnight in a vacuum chamber to obtain the desired compound (7 mg, 3.7 μmol, 77% yield). 1H NMR (400 MHz, DMSO) δ 8.03–7.95 (m, 2H), 7.90 (t, J = 5.7 Hz, 1H), 7.85–7.74 (m, 2H), 7.67 (d, J = 3.8 Hz, 1H), 7.65–7.62 (m, 2H), 7.59–7.52 (m, 2H), 7.51–7.45 (m, 1H), 7.40–7.26 (m, 7H), 6.50 (d, J = 7.1 Hz, 2H), 5.88–5.78 (m, 2H), 5.01–4.86 (m, 4H), 4.63–4.45 (m, 6H), 4.10–4.02 (m, 2H), 3.99 (t, J = 6.0 Hz, 2H), 3.76 (t, J = 5.5 Hz, 2H), 3.73 (s, 3H), 3.47 (broads, 60H), 2.96–2.88 (m, 5H), 2.27 (t, J = 6.0 Hz, 2H), 2.20–2.13 (m, 2H), 2.10 (s, 3H), 2.09 (s, 3H), 2.03–1.91 (m, 3H), 1.79–1.66 (m, 2H), 1.57–1.44 (m, 1H), 1.37–1.31 (m, 1H), 1.28–1.22 (m, 6H). MALDI-TOF MS Calculated for C_92_H_129_N_19_O_24_ [M + H]^+^: 1884.9458, found 1884.150.

### In Vitro Reporter Cell Assays.

Cell reporter assays were utilized in THP1-Dual and A549-Dual cell lines, as adapted from the manufacture protocols. Briefly, cells were plated at a density of 50,000 cells/well in a total volume of 180 μL of supplemented media in cell-culture treated 96-well plates overnight. After 24 h, cells were dosed with 20 μL of treatment groups (for a total volume of 200 μL/well in a 10:1 dilution, with either a 1:1 or 2:1 dilution down the plate) overnight. After 24 h of treatment, cells were pelleted at 1500 RPM for 5 minutes in the centrifuge, and 20 μL of supernatant was plated in a white-walled 96-well plate for analysis by QUANTI-Luc^™^ (InvivoGen) assay. After loading in a plate reader, 50 μL of QUANTI-Luc reagent was added to each well and luminescence was measured for determination of cell-based activity. To the remaining cells in the cell-culture treated 96-well plate, 30 μL of Cell-Titer Glo reagent (Promega) was added and the plate was incubated at 37 °C for 1 h. After incubation, the plate was loaded into the plate reader and luminescence was measured to determine cell-mediated toxicity. Data were recorded in triplicate and analyzed in GraphPad PRISM (Version 10), with data reported with standard error of the mean (SEM).

### In Vitro BMDC/BMDM Maturation and Activity.

Bone marrow primary cells were harvested from both the femur and tibia of female C57BL/6 mice, aged between 6–8 weeks. After harvesting, cells were flushed with cold 1x PBS, centrifuged at 1500 rpm for 5 min, and resuspended in complete media (RPMI 1640 supplemented with 10% HI-FBS (Gibvo), 100 U ml − 1 penicillin, 100 μg ml − 1 streptomycin (Gibco), 2 mM L-glutamine, 10 mM HEPES, 1 mM sodium pyruvate, 1x non-essential amino acids, and 50 μM ß-mercaptoethanol. 20 ng/mL GM-CSF was added to culture BMDCs, and 20 ng/mL of M-CSF was added to culture BMDMs. A single cell suspension was generated by passing the collected cells through a 70 μm sterile cell strainer (Fisherbrand^™^; Thermo Fisher Scientific) and cells were then plated in non-tissue-culture-treated petri dishes (REF 351029; Corning) and incubated at 37°C with 5% CO_2_. Cells were provided with fresh culture media – supplemented with growth factors as described above – on days 3, 5, and 7. On day 8, the cells were collected and confirmed for either CD11c^+^ expression (BMDCs) or CD11b^+^F4/80^+^ expression (BMDM) using flow cytometry with fluorescent anti-CD11c (Clone N418; BioLegend), anti-CD11b (Clone M1/70, BioLegend), and anti-F4/80 (Clone BM8, BioLegend) antibodies. Primary cells were seeded into 12-well plates for analysis by qPCR or 96-well plates for *in vitro* flow cytometry.

### Quantitative RT-PCR (qPCR).

RNA was extracted either from animal tissue (by TissueLyser II, Qiagen) or from in vitro cell cultures using the RNeasy^®^ Plus Mini Kit (Qiagen) according to the manufacturer’s protocol. cDNA was generated through a reverse transcriptase reaction using the iScript cDNA synthesis kit (Bio-Rad), by following the manufacturer’s instructions. To run the qPCR, cDNA was mixed with TaqMan gene expression kits (primer and master mix) to a final volume of 20 μL and run on the Bio-Rad CFX Connect Real-time System, with a threshold cycle number determination made by the Bio-Rad CFX manager software V.3.0. Primers used included: mouse *Ifnb1* (Mm00439552_s1), mouse *Tnf* (Mm00443258_m1), mouse *Cxcl10* (Mm00445235_m1), mouse *Cxcl1* (Mm04207460_m1), and mouse *Hmbs* (Mm01143545_m1). Gene expression was first normalized to the housekeeping gene, *Hmbs*, and then normalized to the PBS treatment within groups using the 2^− ddCt^ analysis method.

### Colocalization Analysis.

A density of 10 × 10^3^ EMT6 and RAW 264.7 cells per well was plated in a glass bottom 96-well plate. After culturing for 12 h, cells were treated for 1.5 h. nAlb-Cy5 (2 μM) and nGFP-Cy5 (2 μM) were added to each well and further incubated for 4 h. Cells were treated with 50 nM of Lysotracker Green (Invitrogen) and 2 μM Hoechst (Invitrogen) for 10 min after the end of the incubation period. Wells were washed three times with phenol red free medium and visualized under confocal microscope (Zeiss LSM880). High-magnification images were obtained using the 40x objective lens. Manders’ coefficient calculated by using Image J Software for colocalization analysis.

### Flow cytometry for In Vitro Uptake Studies.

EMT-6 and RAW 264.7 cells were seeded in 6 well-plates at 5×10^5^ cells per well. After 24 h, cells were treated with and without 5-(N-Ethyl-N-isopropyl) amiloride (EIPA, 50 μM) for 1.5 h. nAlb-Cy5 (2 μM) and nGFP-Cy5 (2 μM) were added to each well and further incubated for 4 hours. EMT6 Cells were digested with 0.25% trypsin. RAW 264.7 cells were collected by scraping. Both cells were washed three times with cold 1x PBS (1ml), and stained with DAPI for live/dead staining. Cells were washed and suspended in a Staining buffer (1x PBS, 2.5% FBS, 2.5 mM EDTA), and detected by flow cytometry. Bone marrow-derived macrophages (BMDMs) were isolated from 6- to 8-week-old female C57BL/6 mice. Cells were collected and BMDMs (CD11b+/F4/80+) were confirmed by flow cytometry. Fluorescence acquisition was carried out on Cytek Aurora spectral flow cytometer and analyzed on FlowJo V.10.8.1.

### Evaluation of Nanobodies in Tumor Models.

For B16.F10, B16.F10-LUC, or B16.F10-OVA tumor models, 6–8 week old C57BL/6 mice (The Jackson Laboratory) were used. For EMT6 tumor models, 6–8 week old Balb/C female mice (The Jackson Laboratory) were used. Tumors were generated in B16.F10 and B16.F10-OVA models by subcutaneous injection of 5 × 10^5^ cancer cells, suspended in 100 μL of PBS, at the right flank of the mouse. B16.F10-LUC inoculations were performed by intravenous (I.V.) injection at a volume of 100 μL and using 1 × 10^6^ cancer cells. EMT6 inoculations were orthotopic and placed at the left-side 4th mammary fat pad at a volume of 100 μL and using 5 × 10^5^ cancer cells. When the volume of tumors reached ~ 75–100 mm^3^, mice were treated by I.V. injection of nanobodies or free diABZI compound 3 (using 40% PEG400 as an excipient for free diABZI, Sigma), or intraperitoneal (I.P) injection of commercial anti-PD-L1 IgG (Clone BE0101, Bio X Cell) (100 μL per injection). For MDSC inhibition studies with the SX-682 inhibitor (CXCL1/2 inhibitor, MedChemExpress), control high fat chow and SX-682 formulated chow (Research Diets, Inc.) were fed to mice at day 4 after tumor inoculation and continued through the course of the study. For studies evaluating the effects of MDSC, NK cell, CD4^+^ T cell, and CD8^+^ T cell depletion, mice (n = 6–8/group) with either EMT6 or B16.F10 tumors were intraperitoneally administered anti-Ly6G/Gr-1 (RB6–8C5; 200 μg), anti-asialo-GM1 (Poly21460; 100 μL for EMT6), anti-NK1.1 (PK136; 200 μL for B16.F10), anti-CD4^+^ (YTS-191; 200 μg), or anti-CD8^+^ (2.43; 200 μg) antibodies one day prior to each treatment with nanobody conjugates. Tumor volume calculations were calculated using V_tumor_ = L × W^2^ × 0.5, in which V_tumor_ is tumor volume, L is tumor length, and W is tumor width. Tumor volume, total murine mass, and murine well-being were recorded for the duration of the study. The endpoint for maximum tumor volume (i.e. survival) during studies was 1500 mm^3^.

### Nanobodies in Spontaneous Tumor Model Studies.

A cohort of female *FVB/N-Tg (MMTV-PyVT)*^*634Mul*^ mice, bred in house, was used for these studies. Study animals were weighed, and mammary glands palpated twice weekly starting at 6 weeks of age. Tumor diameters in two dimensions were obtained using calipers. Treatment for the full cohort was initiated when first palpable tumors appeared at approximately 8–10 weeks of age. The mice received the nanobody-STING agonist conjugate or vehicle control for a total of 3 treatments at 7 day intervals. The study was terminated 22 days after the first treatment. At necropsy, tumors were removed and a wet weight obtained for each. One tumor was fixed in 10% buffered formalin for histological analysis. All tumor measurements and analysis were performed by individuals blinded to treatment group.

### Adoptive OT-I T Cell Transfer in B16.F10-OVA Tumor Model.

CD45.1^+/−^ OT-I mice were a gift of Y. Kim at Vanderbilt University Medical Center. 6–8 week C57BL/6 CD45.1^+/−^ OT-I mice were euthanized and spleens were harvested using EasySep Mouse CD8^+^ T Cell Isolation Kit (STEMCELL Technologies). Briefly, T cells were activated in vitro in supplemented RPMI 1640 (Gibco) with 10% HI-FBS (Gibco), 1% penicillin/streptomycin (Gibco), 50 μM ß-mercaptoethanol (MilliporeSigma), 1 mM sodium pyruvate, minimum essential medium NEAA (non-essential amino acids) (Gibco), 10 mM HEPES (Gibco), recombinant mouse interleukin-2 (10 U/ml; MilliporeSigma), and Dynabeads Mouse T-Activator CD3/CD28 (at a bead-to-cell ratio of 1:1; Gibco) at 37 °C in a CO_2_ incubator (5%). After 5 days, T cells were magnetically separated from Dynabeads and allowed to rest for 24 h before use. The following day, in the B16.F10-OVA model, 5 × 10^5^ OT-I CD8^+^ T cells were adoptively transferred by retro-orbital injection.

### Western Blot Analysis.

Mice were euthanized and tumors (EMT6 and B16.F10) were harvested and 500 μl RIPA buffer (Sigma) supplemented with protease inhibitors (Sigma) was added in approximately 10 mg of tissue. Tissue was homogenized using a bead mill tissue homogenizer (TissueLyser II; Qiagen) and kept the on ice for 30 min. For *in vitro* analysis of STING expression in EMT6 cells were incubated in RIPA buffer for 10 minutes on ice. Protein concentration was measured using a BCA protein assay kit (Thermo Scientific). Equal amount of protein (30 μg) was subjected to SDS-PAGE and transferred onto nitrocellulose membranes using the semi-dry transfer protocol (Bio-Rad). After transfer, membranes were probed with each respective primary antibody (anti-SPARC, anti-TBK1, anti-p-TBK1, anti-sting, anti Hsp-90 and anti-β-actin) overnight at 4°C. Following incubation, the membranes were probed with HRP-conjugated secondary antibodies. All antibodies were purchased from Cell signaling. Protein bands were visualized using ECL western blotting substrate (Thermo scientific). Images of immunoblots were obtained using a LI-COR Odyssey Imaging System.

### Immunofluorescent analysis of EMT6 Tumors.

5-micron Paraffin-embedded tissue sections were prepared for immunofluorescence and stained with anti-CD31 (cell signaling #77699; 1:500), anti-SPARC (cell signaling #5420, 1:500), and anti-CD45 (cell signaling #70257; 1:500). Tissue slides were deparaffined in xylene and rehydrated in serial ethanol dilutions. Antigen retrieval was performed by heating slides for 17 minutes in Tris EDTA buffer, pH 9 in a pressure cooker at 110 °C. Slides were cooled to room temperature and then blocked with 2.5% horse serum (vector labs). After blocking, slides were incubated overnight at 4 °C with primary antibody in horse serum. Slides were then incubated in anti-rabbit HRP secondary (vector labs) for 1 h at room temperature the following day and subsequently incubated in 1:500 Opal 520 (green) or Opal 570 (red) (Akoya) for 10 minutes. For serial staining, slides were stripped using Citric Acid buffer, pH 6.1 in a pressure cooker at 110 °C for 2 minutes and then staining was repeated using different antibody and Opal fluorophore. After the last Opal staining, slides were mounted using antifade gold mount with DAPI (Invitrogen). Stained images were acquired using a Keyence digital microscope system. Images were analyzed with Fiji software. Quantification of markers was done by measuring total amount of fluorescence divided by total number of cells (DAPI).

### Flow Cytometric Experiments and Analysis.

EMT6 tumor bearing Balb/c and B16.F10-OVA bearing C57BL/6 mice were euthanized either 24 h or 48 h after final treatment. Spleens and tumors were harvested, weighed, and placed on ice. Tumors were digested in RPMI 1640 media containing a tumor dissociation kit (collagenase III and deoxyribonuclease I, Miltenyi Biotech). Tumors were further dissociated using an OctoMACS separator (Miltenyi Biotech) and incubated for 30 min at 37 °C for complete digestion. Tumors and spleens were mashed and separated into single cell suspensions using a 70 μm cell strainer (Fisherbrand^™^; Thermo Fisher Scientific) and red blood cells were lysed twice using ACK lysis buffer (Gibco). Cells were resuspended in flow buffer (1x PBS supplemented with 2% FBS and 50μM dasatinib), counted, and stained with Fc-block (aCD16/32, 2.4G2, Tonbo) for 15min at 4 °C, and then stained with the appropriate antibodies for 1hr at 4 °C (found below and in **Tables S1-S2**). After staining, cells were then washed again with FACS buffer, fixed with 2% paraformaldehyde for 10min, washed again with FACS buffer containing AccuCheck counting beads, and analyzed on a Cytek Aurora flow cytometer. All flow cytometry data were analyzed using FlowJo software (version 10; Tree Star; https://www.flowjo.com/solutions/flowjo). Representative flow cytometry plots and gating schemes are shown in **Fig. S34–41.**

### Antibodies for Immune Cell Memory in B16.F10-OVA Tumor Model.

The antibodies used were eFluor 780 viability dye (eBioscience), anti-CD3e (145–2C11, BV510, BioLegend), anti-CD8a (KT15, FITC, Invitrogen), anti-CD4 (RM4–5, violetFluor^™^ 450 Anti-Mouse CD4, FisherScientific), anti-CD69 (H1.2F3, PE/Cy7, BioLegend), anti-CD44 (IM7, PE/Cy5, BioLegend), and anti-CD62L (MEL-14, BV711, BioLegend), and PE-labeled pOVA/H-2Kb tetramer.

### Pharmacokinetics and Ex Vivo Imaging Experiments.

Healthy (Balb/c or C57BL/6) and EMT6 tumor bearing (Balb/c) mice were injected with 100 μL of Cy5 (either as free dye or as a nanobody conjugate) at a dose of 2 mg/kg intravenously. For pre-treatment with the diABZI conjugated nanobody, a dose was prepared at 1.25 μg diABZI in 100 μL total and injected 3 days prior to Cy5 dosing. Blood draws were taken using heparinized capillary tubes (DWK Life Sciences) at discrete time points up to five days after injection. 1 μL of blood was mixed with 50 μL of PBS, centrifuged, and the diluted plasma was collected for analysis. Prescence of Cy5 was determined by fluorescence intensity using a plate reader, with an excitation wavelength of 645 nm and an emission wavelength of 675 nm. Pharmacokinetic analysis was performed in GraphPad Prism (V10) using either a one-phase decay or two-phase decay, in which the reported half-life is the second phase (elimination). Biodistribution studies were performed by excising and weighing hearts, lungs, livers, spleens, kidneys, and tumors. Tissues were washed in 1x PBS and transferred to the stage of the IVIS Lumina III (PerkinElmer). After IVIS, tissue were homogenized using cell disruption in a volume of 200 μL 1x PBS. Homogenized tissue were centrifuged and the supernatant containing the Cy5 dye was quantified for the tissue was determined by fluorescence intensity using a plate reader. A standard curve was generated of free DBOC-Cy5 dye in 1x PBS and concentrations of Cy5 in tissue were calculated by fitting the standard curve to a linear regression. Fluorescence (radiant efficiency) was measured with a maximum value of 1.56 × 10^10^, and a minimum of 8.21 × 10^8^, and areas were drawn manually for organs to generate average radiant efficiency values (per cm^2^) using the Living Image software (version 4.5). For B16.F10-LUC studies, lungs were placed in black 12-well plates (Cellvis) and incubated for 5 min in a solution of 1 mg/mL Pierce^™^ D-Luciferin, Monopotassium Salt (Thermo Fisher Scientific) in 1x PBS. Images were taken on the IVIS and luminescence was quantified as total radiant flux (p/s) for each set of lungs

### Serum Analysis for Anti-VHH Antibodies.

Mice were pre-treated with PBS or nAlb-diABZI (1.25 μg dose of diABZI) three times every three days, or treated once with nAlb-diABZI (1.25 μg dose of diABZI). 14 days after the first dose, blood was collected by cardiac puncture in and allowed to clot to extract serum. Tubes were centrifuged at 2000 × g for 15 min at 4 °C, and the serum was then collected and diluted directly in PBS (1:4 to 1:8192) for analysis. MonoRab rabbit anti-camelid VHH antibody plates (GenScript) were used to determine anti-VHH antibodies in mouse serum. 3 μg in 100 μL of anti-albumin nanobody were loaded into each well of the 96 well plate and allowed to incubate in the pre-coated antibody plate, sealed, and incubated at 37 °C for 30 minutes. The plate was washed with 200 μL of PBST four times. Either the diluted mouse serum or a commercial Rabbit anti-Camelid VHH antibody (Genscript; A01860) were added in serial dilutions to the wells of the plate at a volume of 100 μL. The plate was sealed and incubated at 37 °C for 30 minutes, followed by washing four times with 200 μL of PBST. A commercial secondary Goat anti-Mouse IgG-FITC conjugate (Invitrogen; 31547) or secondary anti-Rabbit IgG-FITC conjugate (Sigma; F9887) was added to the mouse serum or commercial anti-VHH, respectively, at 100 μL and incubated for 30 minutes at 37 °C before washing with PBST four times (200 μL). The plate was quantified using the fluorescence intensity of FITC (ex: 495 nm, em: 515 nm) using a plate reader.

### Ex VivoPlasma Analyte Analysis.

Blood was collected by either cheek bleed or cardiac puncture in K_2_EDTA-coated tubes (BD Biosciences). Tubes were centrifuged at 2000 × g for 15 min at 4 °C, and the plasma was collected for analysis. Cytokine levels were evaluated using either the LEGENDplex^™^ Mouse Anti-Virus Response Panel (BioLegend) or the LEGENDplex^™^ Mouse Cytokine Panel 2 (BioLegend), both with V-bottom plates, according to manufacturer’s instructions, and data were collected using flow cytometry. Cytokine concentrations were interpolated from standard curves using an asymmetric sigmoidal 5-paramater logistic curve fits (GraphPad Prism V10). Bar plots comparing groups and heat maps of averaged values for groups were generated to analyze results.

### NanoString nCounter Analysis of EMT6 Tumors.

After three treatments of nAlb-diABZI (1.25 μg, n = 3–4), AP-diABZI (1.25 μg, n = 3–4), or PBS (n = 3–4) in EMT6 bearing female Balb/c mice, tumors were isolated, digested, and 100 ng of RNA was isolated, as described in the qPCR section. RNA was hybridized to the IO360 PanCancer panel, as well as through a selected gene panel, of target-specific fluorescent barcodes and analyzed using NanoString nCounter MAX Analysis system. The fold change for genes within groups was calculated by comparing against the average normalized gene expression values within PBS treated mice. All statistical significance, and clustering analysis, was performed in R (http://cran.r-project.org) based on the genes provided in the IO360 PanCancer panel.

### Safety Statement.

All research performed in this study was done so with careful consideration of any risks that are inherent to the materials, instruments, and experiments performed. All research safety guidelines and considerations as provided by the safety data sheets (SDS) and university guides were adhered to for the duration of this study.

### Statistics.

All data were plotted and statistical analysis performed using Prism 10 (GraphPad) software. Unless indicated in the figures, all data are presented as mean ± SEM. For comparisons between two groups, unpaired two-tailed Student’s t-tests were performed as indicated. For multiple comparisons a one-way ANOVA was performed with post-hoc Tukey’s correction for multiple comparisons. For tumor volume, statistically significance was examined through a two-way ANOVA followed by Tukey’s adjustment for multiple comparisons. A Log-rank (Mantel-Cox) test was used to compare Kaplan-Meyer survival data.

## Data Availability

All materials used or generated in this study are available to researchers following appropriate standard material transfer agreements. Data are available upon request.
